# Trends and inequalities in the nutritional status of adolescent girls and adult women in sub-Saharan Africa since 2000: a cross-sectional series study

**DOI:** 10.1136/bmjgh-2020-002948

**Published:** 2020-10-08

**Authors:** Safia S Jiwani, Giovanna Gatica-Domínguez, Inacio Crochemore-Silva, Abdoulaye Maïga, Shelley Walton, Elizabeth Hazel, Barbara Baille, Sujata Bose, William K Bosu, Kofi Busia, Tome CA, Ferima Coulibaly-Zerbo, Cheikh Mbacké Faye, Richard Kumapley, Vrinda Mehra, Serge M A Somda, Roosmarijn Verstraeten, Agbessi Amouzou

**Affiliations:** 1 International Health, Johns Hopkins University Bloomberg School of Public Health, Baltimore, Maryland, USA; 2 International Center for Equity in Health, Universidade Federal de Pelotas, Pelotas, RS, Brazil; 3 Regional Office, UNICEF, Dakar, Senegal; 4 Alive & Thrive/FHI Solutions, Washington, DC, USA; 5 Department of Public Health and Research, West Africa Health Organization, Bobo Dioulasso, Burkina Faso; 6 Department of Healthcare Services, West Africa Health Organization, Bobo Dioulasso, Burkina Faso; 7 Department of Planning and Health Information, West African Health Organisation, Bobo-Dioulasso, Hauts-Bassins, Burkina Faso; 8 Regional Office AFRO, World Health Organization, Brazzaville, Congo; 9 West Africa Regional Office, African Population and Health Research Center, Dakar, Senegal; 10 Data and Analytics Section, Division of Data, Analysis, Planning and Monitoring, UNICEF, New York, New York, USA; 11 Division of Poverty, Health and Nutrition, International Food Policy Research Institute, Dakar, Senegal

**Keywords:** nutrition, public health

## Abstract

**Introduction:**

Evidence on the rate at which the double burden of malnutrition unfolds is limited. We quantified trends and inequalities in the nutritional status of adolescent girls and adult women in sub-Saharan Africa.

**Methods:**

We analysed 102 Demographic and Health Surveys between 1993 and 2017 from 35 countries. We assessed regional trends through cross-sectional series analyses and ran multilevel linear regression models to estimate the average annual rate of change (AARC) in the prevalence of underweight, anaemia, anaemia during pregnancy, overweight and obesity among women by their age, residence, wealth and education levels. We quantified current absolute inequalities in these indicators and wealth-inequality trends.

**Results:**

There was a modest decline in underweight prevalence (AARC=−0.14 percentage points (pp), 95% CI −0.17 to -0.11). Anaemia declined fastest among adult women and the richest pregnant women with an AARC of −0.67 pp (95% CI −1.06 to -0.28) and −0.97 pp (95% CI −1.60 to -0.34), respectively, although it affects all women with no marked disparities. Overweight is increasing rapidly among adult women and women with no education. Capital city residents had a threefold more rapid rise in obesity (AARC=0.47 pp, 95% CI 0.39, 0.55), compared with their rural counterparts. Absolute inequalities suggest that Ethiopia and South Africa have the largest gap in underweight (15.4 pp) and obesity (28.5 pp) respectively, between adult and adolescent women. Regional wealth inequalities in obesity are widening by 0.34 pp annually.

**Conclusion:**

Underweight persists, while overweight and obesity are rising among adult women, the rich and capital city residents. Adolescent girls do not present adverse nutritional outcomes except anaemia, remaining high among all women. Multifaceted responses with an equity lens are needed to ensure no woman is left behind.

**Summary box**

**What is already known?**

- The double burden of malnutrition is common to the poorest countries in low-income and middle-income countries where rapid changes in food systems, diets and physical activity have led to a rise in the prevalence of overweight and obesity.
- However, little is known on the rate at which the double burden of malnutrition has unfolded and the subgroups that are most vulnerable.
- In sub-Saharan Africa, the prevalence of overweight and obesity has increased over the past two decades among urban women, and higher wealth status has been associated with higher overweight and obesity, as well as lower underweight prevalence.
- Anaemia continues to affect 50% of pregnant women in this region, acting as a ‘triple burden’ of malnutrition.

**What are the new findings?**

- Our study provides evidence for a situation of lower underweight, overweight and obesity prevalence among adolescent girls compared with adult women.
- Over the past two decades, anaemia has declined the fastest among adult women and the richest pregnant women, although it remains a threat to all women.
- Overweight and obesity have been rising faster among adult women, as well as women living in the richest households.
- Wealth inequalities in obesity have widened over time in sub-Saharan Africa.

**Summary box**

**What do the new findings imply?**

- Our study calls for multifaceted programmatic and policy responses to address the double burden of malnutrition scenario among sub-Saharan African women.
- An equity-focused approach is needed to tackle socioeconomic and demographic inequalities existing in the nutritional status of women and to ensure no woman is being left behind.

## Introduction

Sub-Saharan Africa (SSA) is home to 12.2% of the world’s women of reproductive age,^1^ and it has historically witnessed the highest prevalence of underweight and anaemia among women and children.^2 3^ Many African countries are off-track in meeting the World Health Assembly Global Nutrition Target of achieving a 50% reduction of anaemia among women of reproductive age by 2025.^4^ Similarly, the achievement of Sustainable Development Goals 2 of ending hunger and all forms of malnutrition is a challenge, particularly with the increase in the number of countries experiencing a double burden of malnutrition (DBM).^5 6^ The latter is characterised by the coexistence of undernutrition with overweight and obesity or diet-related non-communicable diseases, within individuals, households and populations across the life course.^7^ More than one-third of low-income and middle-income countries (LMIC) experience multiple forms of malnutrition, and this scenario is most prevalent in SSA, South Asia, East Asia and the Pacific.^8^

Obesity, which was believed to affect only the ‘elite’ in LMIC,^9^ has doubled globally and tripled in the last two decades in LMIC.^10^ In 2010, overweight and obesity were responsible for 3.4 million deaths globally.^11^ By 2016, 44% of adults were overweight or obese, of which 70% lived in LMIC.^12^ In SSA, rapid urbanisation and economic development have led to a nutrition transition^5 13^ characterised by a progressive shift in eating patterns, diets and lifestyles, leading to up to 50% of urban women being classified as overweight or obese in some countries.^14^Although the rise has been witnessed among children, adolescents and adults,^11^ the burden appears to be unevenly distributed across socioeconomic status and gender: research in this region has pointed to a higher obesity prevalence among women, the rich and urban residents.^15^

The additional anaemia burden and micronutrient deficiencies affecting half of the population of pregnant women in SSA has been referred to as a ‘triple burden’,^10^ further exacerbating the cycle of malnutrition, poverty and infection. Maternal underweight and iron deficiency are associated with an increased risk of low birth weight, deficits in development and child mortality, while maternal overweight and obesity contribute to maternal morbidity, non-communicable diseases and preterm birth.^16^ Furthermore, a quarter of maternal deaths globally result from pre-existing conditions including obesity.^17^ The optimal nutritional status of adolescent girls and women of reproductive age is, therefore, an essential ingredient for a healthy pregnancy and positive maternal and child health outcomes. Adolescence provides a critical window of opportunity for catch-up growth and to break the intergenerational cycle of poor growth.^18^

While several studies have assessed the DBM in the SSA context including the recently published Lancet series on DBM,^5 6 8 10^ there is little evidence on inequalities that exist in the nutritional status of adolescent girls and adult women, and trends within subpopulations, based on socioeconomic and demographic factors. We provide this evidence by (1) assessing the trends of women’s nutritional status by estimating the average annual rate of change (AARC) in underweight, anaemia, anaemia during pregnancy, overweight and obesity among adolescent girls and adult women across 35 SSA countries since 2000 and (2) identifying the subgroups that are left behind by evaluating the current absolute inequality gap in these indicators comparing adolescent girls and adult women, women with varying wealth status, place of residence and education levels, using the latest available data in each country and lastly (3) assessing whether wealth inequalities have been widening or closing over time by quantifying the change in the average annual regional gap in women’s nutritional status between the richest and poorest women.

## Methods

### Study design and data

We conducted a cross-sectional series analysis of the prevalence of underweight, anaemia, anaemia during pregnancy, overweight and obesity among women of reproductive age (15–49 years) by use of 102 nationally representative Demographic and Health Surveys (DHSs) retrieved from 35 countries in SSA between 1993 and 2017. The DHSs are implemented in over 90 LMIC, and collect measured height and weight variables as well as the diagnosis of any type of anaemia among women of reproductive age and during pregnancy; all data are deidentified and made publicly available (https://dhsprogram.com/).

The trends analysis in underweight, overweight and obesity was based on 30 countries that conducted at least two DHS surveys since 1993, whereas 18 and 20 countries had at least two surveys with data on anaemia during pregnancy and anaemia, respectively. Only 8 out of 35 countries had a recent survey since 2015. The analysis of current absolute inequalities in these indicators used the latest survey for each country and was restricted to 31 countries with a survey in the 2010–2017 period.

### Procedures

We calculated body mass index (BMI) as the weight in kilograms divided by the height in metres squared, and defined our outcomes of interest based on WHO BMI cut offs among adult women aged 20–49 years: below 18.5 kg/m^2^ was classified as underweight, 25 kg/m^2^ to below 30 kg/m^2^ as overweight and 30 kg/m^2^ or more as obese.^19^ For adolescent girls aged 15–19 years, we used the standard deviations (SD) cut-offs for median BMI-for-age based on WHO 2007 reference growth: below −2 SD for underweight, between 1 and 2 SD for overweight, and greater than 2 SD for obesity.^20^ Anaemia was reported by haemoglobin levels as mild, moderate, severe or any anaemia, and was adjusted for altitude and cigarette smoking, which are known to increase haemoglobin concentrations.^21^ We did not report estimates when the sample size of the subgroup of women was smaller than 25.

We presented stratified analyses of these indicators by woman’s age (adolescents aged 15–19 years compared with adults aged 20–49 years), woman’s wealth status using the existing wealth quintiles in the DHS surveys computed by principal component analysis and adjusted for urban and rural residence according to DHS methodology^22^ (first quintile being the 20% poorest, and fifth quintile, the 20% richest households), woman’s place of residence (capital city/region or largest city, other urban area, rural area) and woman’s education level (no education, primary, secondary or more). In Côte d’Ivoire, Nigeria and Tanzania, we used the largest commercial city (Abidjan, Lagos and Dar Es Salaam, respectively) instead of the capital city; when the capital city was not isolated in a survey, we used the region that included the capital city instead. For instance, in South Africa, we used the three capital regions of Western Cape, Gauteng and Free State. In Cameroon and Gabon, the surveys combined two cities as the capital: Yaounde and Douala, and Libreville and Port-Gentil, respectively. As a supplementary analysis in the online supplemental appendix figure S2 A–E, we further disaggregated adult women into younger adults (20–34 years) and older adults (35–49 years).

10.1136/bmjgh-2020-002948.supp1 Supplementary data

### Statistical analysis

We assessed trends over time by computing the AARC in nutritional status outcomes as the slope of the multilevel linear regressions of outcome prevalence on survey year, separately for each age, wealth, residence and education subgroup, with survey time point as the first level and country as the second level. A positive AARC value depicts an average annual percentage point (PP) rise in the outcome of interest, whereas a negative AARC value depicts a PP decline. We restricted the analysis to countries that had at least two observations. We ran separate stratified unweighted analyses by woman’s age, wealth, residence and education categories, for the entire SSA region, as well as by subregion (Eastern and Southern Africa (ESA), and Western and Central Africa (WCA)).

We measured current absolute inequalities by calculating the difference in PPs of the prevalence of nutritional outcome between female subgroups, within each country: adults (20–49 years) vs adolescents (15–19 years), 20% richest vs 20% poorest, capital city residents versus other urban city residents and rural residents, etc.

To further understand if wealth-related inequalities in nutritional status are widening or closing over time, we ran the multilevel linear regression model with an interaction between wealth status (20% poorest vs 20% richest) and survey year, to estimate the change in the average annual regional gap in nutritional outcome between the richest and poorest women.

All analyses and graphs were conducted on Stata V.15 (StataCorp). We took into account complex survey weights and sampling designs using the svy command to estimate the prevalence of nutritional outcomes in each subgroup. Maps were generated using Tableau Public 2020.2 (https://www.tableau.com).

### Patient and public involvement

This study was entirely based on secondary analyses of publicly available datasets collected in the countries included in the study. Representatives from the Ministry of Health and research institutions from 15 countries in West Africa were involved in the analysis, interpretation and dissemination of the findings through two analytical workshops conducted as part of the Countdown to 2030 regional initiative for West Africa.

## Results

The trends analyses included a total of 631 288 adolescent and adult women with data on underweight, overweight and obesity, 367 156 adolescent and adult women with anaemia data, and 33 715 pregnant women with anaemia during pregnancy data. The analysis of current inequalities in these indicators covered 237 110 women for underweight, overweight and obesity, 183 169 women for anaemia and 16 737 pregnant women for anaemia during pregnancy. Sample sizes by country are presented in online supplemental appendix tables S1–S5.

### Underweight

**Figure 1:**
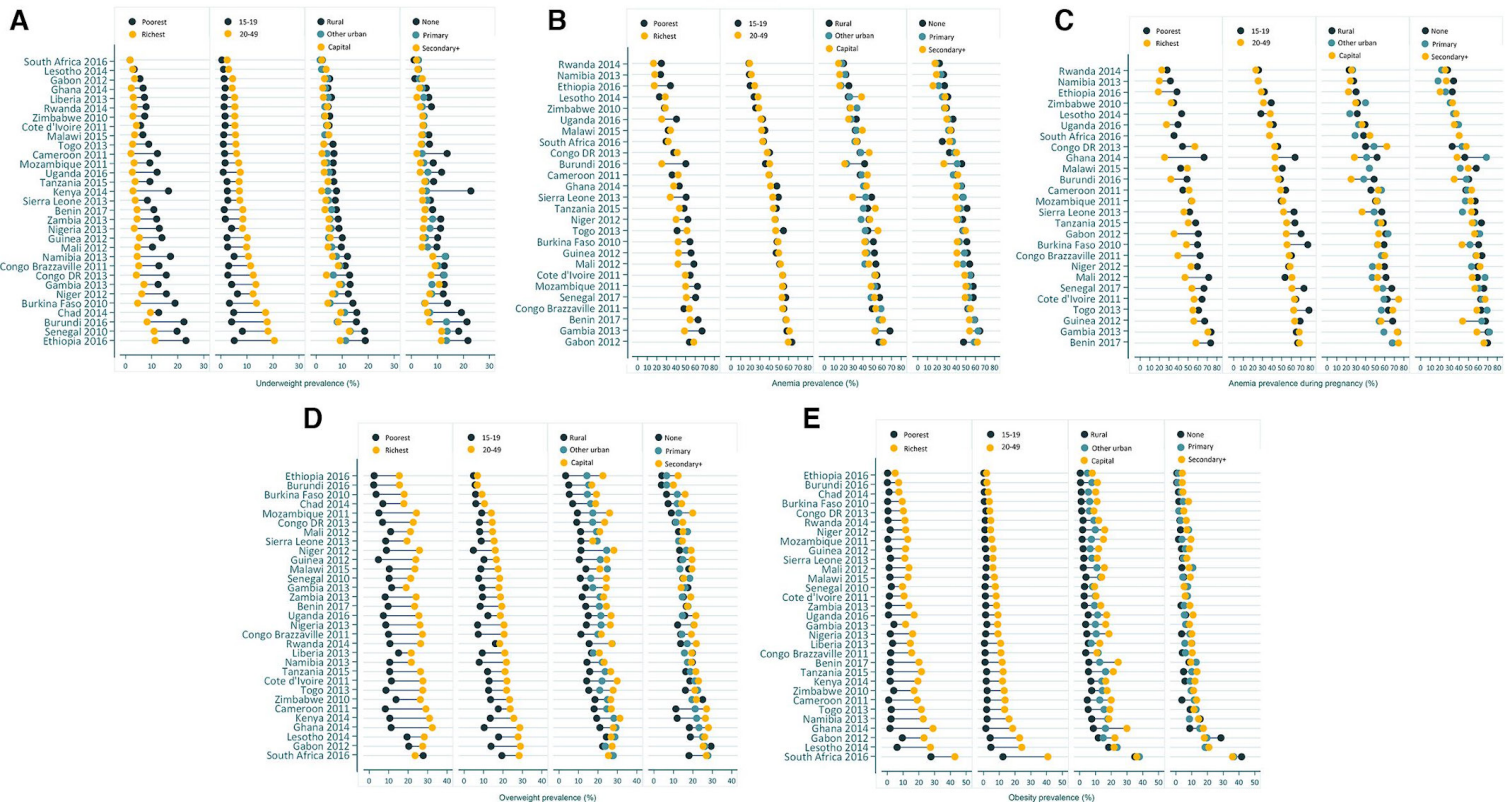
Inequalities in prevalence (%) of underweight (A), anaemia (B), anaemia during pregnancy (C), overweight (D) and obesity (E) by women’s wealth, age, residence and education.

The most recent surveys in SSA suggest that the highest prevalence of underweight among women is observed in Ethiopia (2016) and Senegal (2010) at 17% and 15.8%, respectively. Online supplemental figure S1A in the appendix maps out the distribution of underweight prevalence by country. However, national level estimates mask inequalities that exist between subgroups: figure 1A enables visualisation of marked absolute inequalities in the prevalence of underweight between adult women and adolescent girls, as well as richest and poorest women. Overall, the gaps were relatively narrow in most countries; However, they were remarkable in the countries with the highest burden, like Ethiopia and Senegal. In Ethiopia (2016), the gap by women’s age was of 15.4 pp, with a prevalence of 20.5% among adult women compared with 5.1% among adolescent girls. Burkina Faso (2010) and Burundi (2016) depicted the largest rich-poor gap, with a −14.4 pp and −14.2 pp difference, respectively. Inequalities in underweight by women’s residence and education were apparent in specific countries: −8.7 pp between other urban and rural residents in Burkina Faso, and −17.0 pp between primary and no education levels in Kenya (figure 1A, online supplemental appendix table S1).

**Figure 2:**
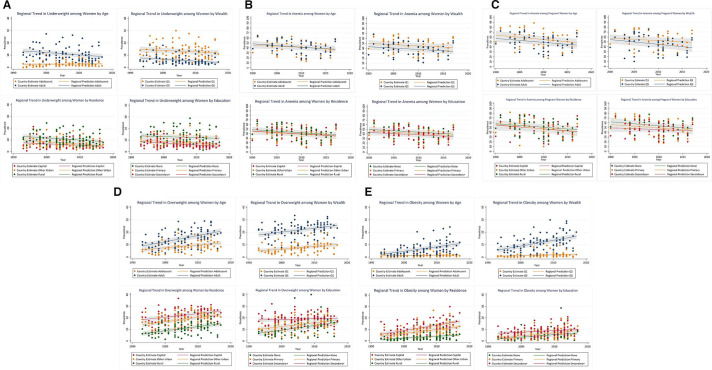
Trend in prevalence (%) of underweight (A), anaemia (B), anaemia during pregnancy (C), overweight (D) and obesity (E) by women’s wealth, age, residence and education.

Figure 2A illustrates the regional trends of underweight among women in the 30 countries with at least two surveys. It suggests that regionally, the prevalence of underweight remains concentrated among adult women (8.8% in 2017), women living in the poorest households (11.9% in 2017), in rural areas (8.6% in 2017) and having no formal education (11.5% in 2017). The slope of the regional trend line depicts the AARC in underweight, listed in table 1, and indicates an overall regional declining trend of −0.14 pp (95% CI -0.17 to -0.11), and declining among all subgroups except adolescent girls, with no significant differences in the decline by women’s residence, wealth or education. A modest annual decline of −0.19 pp (95% CI -0.26 to -0.12) was observed among adult women, whereas there was no change among adolescent girls who had a much lower prevalence of underweight during the same time period (2.9% in 2017).

**Table 1:** Average annual rate of change (AARC) in prevalence of underweight, anaemia, anaemia during pregnancy, overweight and obesity by woman’s age, residence, wealth and education for countries with at least two surveys in the period 1993–2017 (in percentage points†)

	Underweight percentage points (95% CI) n=96 surveys ‡		Anaemia percentage points (95% CI) n=52 surveys§		Anaemia pregnancy percentage points (95% CI) n=39–52 surveys¶		Overweight percentage points (95% CI) n=96 surveys‡		Obesity percentage points (95% CI) n=96 surveys‡	
SSA										
Age										
15–19 years	0.00	(−0.02 to 0.05)	−0.46^*^	(−0.85 to −0.07)	−0.90^*^	(−1.52 to −0.29)	0.23**	(0.15 to 0.31)	0.03	(−0.01 to 0.07)
20–49 years	−0.19**	(−0.26 to −0.12)	−0.67**	(−1.06 to −0.28)	−0.79^*^	(−1.31 to −0.27)	0.49**	(0.42 to 0.56)	0.36**	(0.30 to 0.42)
Residence										
Capital/largest city	−0.13**	(−0.20 to −0.06)	−0.62^*^	(−1.11 to −0.13)	−0.78	(−1.67 to 0.11)	0.31**	(0.20 to 0.42)	0.47**	(0.39 to 0.55)
Other Urban area	−0.19**	(−0.25 to −0.13)	−0.61**	(−0.96 to −0.26)	−0.92^*^	(−1.59 to −0.24)	0.37**	(0.28 to 0.46)	0.34**	(0.28 to 0.40)
Rural area	−0.18**	(−0.24 to −0.12)	−0.65^*^	(−1.08 to −0.22)	−0.73^*^	(−1.24 to −0.23)	0.35**	(0.29 to 0.41)	0.16**	(0.12 to 0.20)
Wealth										
Poorest (Q1)	−0.11^*^	(−0.22 to 0.00)	−0.50^*^	(−0.97 to −0.03)	−0.48	(−1.02 to 0.07)	0.22**	(0.15 to 0.28)	0.06^*^	(0.03 to 0.09)
Richest (Q5)	−0.15**	(−0.20 to −0.10)	−0.55^*^	(−0.90 to −0.19)	−0.97^*^	(−1.60 to −0.34)	0.31**	(0.24 to 0.39)	0.46**	(0.39 to 0.53)
Education										
None	−0.09	(−0.19 to 0.01)	−0.60^*^	(−1.05 to −0.15)	−0.84^*^	(−1.56 to −0.13)	0.39**	(0.28 to 0.50)	0.22**	(0.16 to 0.28)
Primary	−0.14**	(−0.21 to −0.08)	−0.58^*^	(−1.01 to −0.15)	−0.70^*^	(−1.23 to −0.17)	0.33**	(0.24 to 0.41)	0.27**	(0.21 to 0.33)
Secondary or more	−0.17**	(−0.24 to −0.10)	−0.55^*^	(−0.94 to −0.16)	−0.36	(−1.04 to 0.31)	0.06	(−0.04 to 0.16)	0.15**	(0.07 to 0.23)
Total	−0.14**	(−0.17 to −0.11)	−0.56**	(−0.66 to −0.47)	−0.74**	(−0.89 to −0.60)	0.34**	(0.30 to 0.39)	0.24**	(0.20 to 0.27)
ESA										
Age										
15–19 years	0.00	(−0.05 to 0.05)	−0.37	(−0.96 to 0.22)	−0.61	(−1.37 to 0.15)	0.21**	(0.12 to 0.30)	0.03^*^	(0.00 to 0.06)
20–49 years	−0.09^*^	(−0.18 to 0.00)	−0.64^*^	(−1.23 to −0.05)	−0.50	(−1.22 to 0.23)	0.43**	(0.35 to 0.51)	0.32**	(0.26 to 0.38)
Residence										
Capital/largest city	−0.01	(−0.07 to 0.05)	−0.54	(−1.25 to 0.17)	0.25	(−0.77 to 1.28)	0.29**	(0.14 to 0.44)	0.48**	(0.37 to 0.59)
Other Urban area	−0.08^*^	(−0.15 to 0.00)	−0.45^*^	(−0.86 to −0.04)	−0.30	(−1.10 to 0.50)	0.34**	(0.20 to 0.48)	0.31**	(0.23 to 0.39)
Rural area	−0.09^*^	(−0.17 to −0.02)	−0.61	(−1.24 to 0.02)	−0.51	(−1.23 to 0.22)	0.31**	(0.24 to 0.38)	0.16**	(0.12 to 0.20)
Wealth										
Poorest (Q1)	0.08	(−0.04 to 0.20)	−0.37	(−1.10 to 0.36)	−0.35	(−1.18 to 0.48)	0.13**	(0.07 to 0.19)	0.04^*^	(0.01 to 0.06)
Richest (Q5)	−0.09^*^	(−0.16 to −0.02)	−0.38	(−0.79 to 0.04)	−0.14	(−0.83 to 0.54)	0.31**	(0.20 to 0.42)	0.46**	(0.37 to 0.56)
Education										
None	0.09	(−0.06 to 0.25)	−0.59	(−1.28 to 0.10)	−0.57	(−1.70 to 0.56)	0.33^*^	(0.14 to 0.52)	0.19**	(0.12 to 0.26)
Primary	−0.06	(−0.14 to 0.02)	−0.56	(−1.18 to 0.05)	−0.48	(−1.19 to 0.24)	0.32**	(0.24 to 0.40)	0.20**	(0.14 to 0.26)
Secondary or more	−0.08^*^	(−0.14 to −0.02)	−0.33	(−0.86 to 0.20)	−0.03	(−0.81 to 0.75)	0.13	(−0.02 to 0.28)	0.24**	(0.17 to 0.32)
Total	−0.06^*^	(−0.11 to 0.00)	−0.48**	(−0.62 to −0.34)	−0.39**	(−0.58 to −0.20)	0.31**	(0.25 to 0.37)	0.22**	(0.17 to 0.27)
WCA										
Age										
15–19 years	0.03	(−0.02 to 0.08)	−0.53^*^	(−1.02 to −0.04)	−1.02^*^	(−1.92 to −0.12)	0.24**	(0.10 to 0.38)	0.03	(−0.03 to 0.10)
20–49 years	−0.28**	(−0.37 to −0.19)	−0.70^*^	(−1.21 to −0.19)	−1.04^*^	(−1.73 to −0.35)	0.55**	(0.44 to 0.66)	0.39**	(0.28 to 0.50)
Residence										
Capital/largest city	−0.23**	(−0.33 to −0.13)	−0.60	(−1.25 to 0.05)	−1.01	(−2.17 to 0.15)	0.32**	(0.17 to 0.47)	0.45**	(0.33 to 0.57)
Other Urban area	−0.29**	(−0.37 to −0.21)	−0.75^*^	(−1.28 to −0.22)	−1.27^*^	(−2.07 to −0.47)	0.39**	(0.28 to 0.50)	0.36**	(0.26 to 0.46)
Rural area	−0.26**	(−0.36 to −0.16)	−0.68^*^	(−1.25 to −0.11)	−0.92^*^	(−1.58 to −0.25)	0.40**	(0.29 to 0.51)	0.16**	(0.09 to 0.23)
Wealth										
Poorest (Q1)	−0.28**	(−0.44 to −0.13)	−0.61^*^	(−1.19 to −0.04)	−0.63	(−1.30 to 0.05)	0.31**	(0.20 to 0.42)	0.09^*^	(0.03 to 0.15)
Richest (Q5)	−0.21**	(−0.28 to −0.15)	−0.64^*^	(−1.20 to −0.09)	−1.39^*^	(−2.24 to −0.54)	0.32**	(0.21 to 0.43)	0.45**	(0.34 to 0.57)
Education										
None	−0.26**	(−0.35 to −0.17)	−0.60^*^	(−1.15 to −0.05)	−1.00^*^	(−1.87 to −0.13)	0.44**	(0.33 to 0.55)	0.25**	(0.15 to 0.35)
Primary	−0.21**	(−0.30 to −0.12)	−0.57	(−1.16 to 0.01)	0.91^*^	(0.17 to 1.64)	0.34**	(0.19 to 0.48)	0.32**	(0.22 to 0.43)
Secondary or more	−0.24**	(−0.35 to −0.13)	−0.68^*^	(−1.26 to −0.11)	−0.88	(−1.85 to 0.08)	0.00	(−0.14 to 0.14)	0.06	(−0.06 to 0.18)
Total	−0.21**	(−0.26 to −0.17)	−0.65**	(−0.77 to −0.53)	−1.07**	(−1.27 to −0.87)	0.38**	(0.31 to 0.44)	0.25**	(0.21 to 0.30)

*P<0.05, **P<0.001.

†AARC in percentage points: 1 percentage point corresponds to a 1% average increase in the prevalence of the nutritional outcome among the subgroup of women each year.

‡30 countries with two or more surveys in SSA region between 1993 and 2017: 13 ESA countries and 17 WCA countries.

§20 countries with two or more surveys in SSA region between 2000 and 2017: 9 ESA countries and 11 WCA countries.

¶18–20 countries with two or more surveys in SSA region between 2000 and 2017: 7–9 countries in ESA and 11 countries in WCA.

ESA, Eastern and Southern Africa; SSA, sub-Saharan Africa; WCA, Western and Central Africa.

Table 1 enables comparing the trends between subregions, and indicates that WCA had a much larger decline in underweight of −0.21 pp (95% CI -0.26 to -0.17) compared with a decline of −0.06 pp in ESA since 2000, with a significant difference by age: adult women in WCA experienced an AARC of −0.28 pp (95% CI -0.37 to -0.19), three times that of adult women in ESA, compared with a rise, although non-significant, of 0.03 pp (95% CI −0.02 to 0.08) among adolescent girls in WCA.

### Anaemia

Anaemia remains a public health threat for all women in this region, with the prevalence in the latest surveys ranging from 19.2% in Rwanda (2014) to 60.6% in Gabon (2012) (online supplemental appendix table S2). Within each country, the prevalence does not vary widely by age: the largest gap of −8.1 pp between adult and adolescent women was observed in Togo (2013). However, wealth-related inequality gaps in anaemia are larger, reaching −25.1 pp in Burundi (2016) where 50.3% of the poorest women are anaemic compared with 25.2% of the richest women. Within subgroups of women, the highest prevalence of 68.4% and 67.3% was found among rural residents and the poorest women, respectively, in Gambia (2013); Burundi and Gambia also had the widest anaemia gaps between residents of other urban areas and rural areas (figure 1B, online supplemental appendix table S2).

Regionally, the trends in anaemia have been declining steadily in SSA since 2000 with an AARC of −0.56 pp (95% CI −0.66 to -0.47), with no significant differences between subgroups (figure 2B, table 1). Table 1 further confirms a significant decline among all subgroups in SSA, the largest one being among adult women (AARC=−0.67 pp, 95% CI −1.06 to -0.28). Comparing ESA and WCA subregions, we observed larger, significant declines in anaemia in WCA, particularly among women living in other urban areas (AARC=−0.75 pp, 95% CI −1.28 to -0.22), whereas in ESA the decline was smaller in magnitude and only statistically significant among residents of other urban areas (AARC=−0.45 pp, 95% CI −0.86 to -0.04).

### Anaemia during pregnancy

Anaemia was even more prevalent among pregnant women, ranging from 23.4% in Rwanda (2014) to 68.4% in Benin (2017). Overall, it was primarily concentrated among the poorest pregnant women, adolescent pregnant women and pregnant women living in the poorest households (online supplemental appendix table S3, figure 1C). Inequalities between subpopulations were particularly marked in Burkina Faso (2010) and Ghana (2014) where there was a −21.5 pp and −20.9 pp absolute gap between adult and adolescent pregnant women, respectively, whereas in Guinea (2012) the prevalence was similar in both age groups (63.7% and 67.3%, respectively) (online supplemental appendix table S3). The prevalence remains higher among the least socioeconomically advantaged women in the majority of countries, with gaps as large as −41.2 pp between richest and poorest pregnant women in Ghana, except in Cameroon, DRC, Malawi and Mozambique. There is no clear pattern in terms of the difference of the anaemia burden by pregnant women’s place of residence nor by education levels across countries (figure 1C).

The trends analysis in figure 2C and table 1 depict a large declining anaemia trend of −0.74pp (95% CI −0.89 to -0.60) among pregnant women in SSA since 2000, with no significant differences by age, wealth, residence or education throughout the years, shown by the overlapping confidence intervals. Table 1 indeed indicates significant average annual declines in anaemia among all subgroups except pregnant women living in the capital city, and those having a secondary or higher education; the largest decline since 2000 is among the richest pregnant women (AARC=−0.97 pp, 95% CI −1.60 to -0.34), although not statistically significantly different from that among the poorest. In the ESA subregion, the AARC was not statistically significant in any of the subgroups, whereas in WCA the decline in anaemia appears to have been almost threefold larger (−1.07 pp, 95% CI -1.27 to -0.87), reaching up to −1.39 pp per year among pregnant women living in the richest households.

### Overweight

In the most recent surveys, overweight was most prevalent among women in South Africa (2016, 26.8%), Gabon (2012, 25.7%), Lesotho (2014, 25.6%) and Ghana (2014, 25.3%). In general, the prevalence was highest among the richest women, adult women, and those living in the capital city. The largest absolute gap was observed in Ghana by women’s wealth (21.2 pp) and by age (18.3 pp). In terms of inequalities by residence, there is a 19.3 pp gap between capital city and other urban dwellers in Sierra Leone, and 13.2pp between urban and rural dwellers in Nigeria. Inequalities in overweight are less pronounced by education levels, where there is a 7.1 pp gap between women with secondary and primary education in Sierra Leone, and a 10.0 pp gap comparing women with primary and no education in Chad (online supplemental appendix table S4, figure 1D).

At regional level, figure 2D and table 1 depict an increasing annual trend of 0.34 pp (95% CI 0.30, 0.39) in overweight since 2000, and it is rising in all groups except women with secondary or more education among whom it is not statistically significant. Specifically, adult women had the highest AARC of 0.49 pp (95% CI 0.42, 0.56) compared with 0.23 pp (95% CI 0.15 to 0.31) among adolescent girls; the AARC in overweight is similar in all three residence groups, higher among the wealthiest women (0.31 pp) and among women with no education (0.39 pp), which is significantly different from the situation among women with secondary or more education (0.06 pp). When comparing subregions, a similar scenario holds in both ESA and WCA, with the largest rise in overweight observed among adult women with a statistically significant AARC of 0.43 pp and 0.55 pp, respectively (table 1).

The supplementary analysis presented in online supplemental appendix figure S2D allowed a better understanding of changing trends in the nutritional status of adult women specifically, by further stratifying adult women into young adults 20–34 years and older adults 35–49. The results indicated that older adult women appear to bear the brunt of the overweight burden since 2000.

### Obesity

Online supplemental appendix table S5 indicates that, in the most recent surveys, obesity was most prevalent in South Africa (2016) where one in three women was obese (36.1%, 95% CI 33.8% to 38.5%), followed by Lesotho (2014) (20.0%, 95% CI 17.9% to 22.0%). Specifically, in South Africa, the prevalence of obesity was over 30% among all subgroups, except adolescent girls and women living in the poorest households. Figure 1E suggests that the prevalence of obesity differs substantially between subgroups: South Africa also had the largest absolute inequalities in obesity between adult and adolescent women (28.5 pp): 40.8% of adult women were obese compared with 12.3% of adolescent girls. Inequalities by wealth status indicate that in Ghana (2014), women living in the richest households had a 27.2 pp higher obesity prevalence compared with women in the poorest households. Similarly, inequalities by residence suggest a 13.6 pp difference between women living in the capital city and those in other urban areas in Ghana (2014), and a 10.8 pp difference between women living in urban compared with rural areas in Tanzania (2015). Obesity affects women regardless of their education levels, with inequalities by education remaining small: 5.8 pp between secondary and primary school attendees in Namibia (2013), and 7.8 pp between primary and no education levels in Cameroon (2011) (online supplemental appendix table S5, figure 1E).

The trend in obesity among women over time illustrated in figure 2E and table 1 indicates a regional annual rise of 0.24 pp (95% CI 0.20 to 0.27) in SSA; the most rapid rise in obesity is among adult women (AARC=0.36, 95% CI 0.30 to 0.42), women living in the richest households (AARC=0.46 pp, 95% CI 0.39 to 0.53), and in the capital city (AARC=0.47 pp, 95% CI 0.39 to 0.55) or other urban areas (AARC=0.34 pp, 95% CI 0.28 to 0.40), thus fueling inequalities over time by age, wealth and residence. These data suggest a threefold more rapid rise in obesity among capital city compared with rural dwellers. The scenario among adolescent girls (AARC=0.03 pp, 95% CI −0.01 to 0.07) and women living in the poorest households (AARC=0.06 pp, 95% CI 0.03 to 0.09) appears much different, with a very mild rise in obesity over time. The situation is similar for both the ESA and WCA subregions, with significant differences in the AARC of obesity by age, wealth and between capital city/other urban residents and rural residents within each subregion.

The supplementary analysis comparing the trend between older and younger adults in SSA suggests a larger prevalence of obesity among older adults, with widening inequality over time (online supplemental figure S2E).

Finally, the trend analysis of wealth inequalities over time presented in table 2 confirmed a statistically significant widening of the wealth inequality gap in obesity by 0.34 pp (95% CI 0.21 to 0.47) annually between richest and poorest women in the SSA region on average, whereas there was no significant change in the inequality gap for underweight, anaemia, anaemia during pregnancy or overweight between richest and poorest women since 2000.

**Table 2:** Trend in wealth-inequalities in prevalence of underweight, anaemia, anaemia during pregnancy, overweight and obesity (1993–2017)

	Underweight percentage points n=96 surveys †		Anaemia percentage points n=52 surveys‡		Anaemia during pregnancy percentage points n=39–52 surveys§		Overweight percentage points n=96 surveys †		Obesity percentage points n=96 surveys †	
Interaction term* β (95% CI)	−0.03	(−0.16 to 0.09)	0.01	(−0.59 to 0.62)	−0.26	(−1.06 to 0.55)	0.01	(−0.10 to 0.13)	0.34**	(0.21 to 0.47)

*Interaction term of multilevel linear regression coefficient interpreted as the difference in the nutritional outcome between the richest and poorest women per year, in percentage points,**P<0.001.

†30 countries with two or more surveys in SSA region between 1993 and 2017: 13 ESA countries and 17 WCA countries.

‡20 countries with two or more surveys in SSA region between 2000 and 2017: 9 ESA countries and 11 WCA countries.

§18–20 countries with two or more surveys in SSA region between 2000 and 2017: 7–9 countries in ESA and 11 countries in WCA.

ESA, Eastern and Southern Africa; SSA, sub-Saharan Africa; WCA, Western and Central Africa.

## Discussion

Regionally, our results from SSA indicate a situation of double burden with a declining yet persisting underweight burden among women while overweight and obesity rise at unprecedented rates in this region, particularly among adult women, those living in the richest households, in other urban areas and capital cities. Our results also indicate that adolescent girls appear not to bear the brunt of the underweight, overweight and obesity burden. However, we found that anaemia remains a persisting threat to both adolescent girls and adult women in the region, further exacerbating the double burden scenario and acting as a third layer of malnutrition.

Our trends analyses point to a small although significant annual decline in underweight among all women except adolescents and women with no formal education, with regional disparities remaining large in 2017 by women’s age and wealth. On the other hand, although the prevalence of anaemia has decreased over time, it remains a serious public health threat to all women in the region, with no marked inequalities between subgroups. In terms of overweight, we found a significant annual rise among all subgroups except women with secondary or higher education: adult women and women living in the richest households bear the brunt of the burden in 2017 with 20.7% and 25.4%, respectively, classified as overweight compared with 11.3% among adolescents and 10.2% among women living in the poorest households, regionally. We also found that overweight is rising at a similar rate among women living in capital, urban and rural areas. Obesity, however, is rising fastest among adult women (AARC=0.36 pp), capital city dwellers (AARC=0.47 pp) and the richest women (AARC=0.46 pp), whereas the rise among adolescent girls is negligible.

Our analyses of absolute inequalities between subgroups showed that within the region, some countries experienced larger burden and wider disparities in nutrition outcomes between subpopulations. In general, we found large absolute inequalities in overweight and obesity by women’s wealth status and the wealth-gap in obesity has widened over time. Underweight, overweight and obesity prevalences were systematically higher among adult women. Specifically, Burundi (2016) and Ghana (2014) had the largest disparities in anaemia and anaemia during pregnancy, respectively, between richest and poorest women. South Africa had both the highest obesity prevalence in all subgroups, as well as the widest absolute inequality gaps in obesity by age (28.5 pp), whereas the largest obesity gaps between capital city and rural dwellers was witnessed in Ghana (2014).

Our results complement The Lancet Series 2019 on the DBM that identified 29 countries in SSA, out of 126 LMIC with country-level DBM, and found that this situation predominates particularly in the poorest LMIC in SSA and South and East Asia. Furthermore, there is increasing evidence on associations between DBM and non-communicable diseases,^23 24^ inflammation and childbirth outcomes.^24 25^ The series called for a double duty approach to addressing DBM that, instead of acting on undernutrition or overnutrition in two distinct silos, aims to simultaneously reduce the risk of both forms of malnutrition by 2030.^26^

Our results are also in line with analyses conducted by Amugsi *et al* using survey data from 24 African countries between 1991 and 2014, reporting an increase in overweight and obesity among women in all countries, with the obesity prevalence tripling in Ghana, Zambia, Burkina Faso, Mali and Tanzania since 1991.^5^ Similarly, another study estimated a six PP rise in obesity among women aged 15–49 years in SSA, using survey data from 1993 to 2014.^27^ Associations between socioeconomic status and obesity among women have been previously reported, suggesting that obesity is more prevalent among the richer households in SSA, although the results by education levels remain inconsistent.^1^,^5^

Rapid rises in overweight and obesity have been observed in LMIC in response to changes in food systems, lifestyles, physical activity urbanisation, migration and countries’ economic development,^8 28 29^ leading to an increased consumption of energy dense foods and reduced physical activity.^12^ Per capita income increases appear to shift the obesity burden towards rural residents and the poor, with over half of the global obesity rise occurring among rural residents.^12^ Recent evidence from the Latin American and Caribbean context similarly suggests a changing obesity burden according to countries’ level of economic development, and an unequal distribution of the epidemic, with the burden increasing faster among lower socioeconomic groups and rural populations, while remaining higher overall among urban residents.^30^ In SSA, the situation appears to be different, with the prevalence of obesity being greater among wealthier households and concentrated heavily in urban areas,^8 28 31^ as suggested by our analysis, thus suggesting that a shift towards lower socioeconomic groups has not yet occurred. This pattern has been described by Jaacks *et al* as the first stage of the obesity transition, characterised by a higher prevalence of obesity among adult women and in higher socioeconomic groups, and common to the SSA context.^32^ Our analyses further point to the increased obesity burden specifically among women living in capital cities, as well as among adult women compared with adolescent girls. Interestingly, our results also indicate that the rate at which overweight has increased since 2000 is similar across urban and rural residents, suggesting that although overweight is concentrated among urban women, it has been rising as fast among rural counterparts, whereas this trend was not observed for obesity.

In recent years, much progress has been made in the SSA region to improve adolescent girls’ and adult women’s nutrition. Programmes in areas of safe water access, malaria prevention, improved sanitation, food security, food fortification, iron-folate supplementation and consumption of diversified local foods have been put in place to reduce the burden of undernutrition and micronutrient deficiencies.^33 34^ In Ghana for instance, the Girls’ Iron-Folate Tablets Supplementation national programme, implemented since 2019, has been successful at providing weekly iron-folate supplements to adolescent girls through schools and other channels.^33 35^ However, our analyses indicate that anaemia remains a persisting threat to all women in SSA, particularly during pregnancy, and this merits attention. The literature indicates that underlying causes of anaemia among women of reproductive age are multifactorial, including insufficient dietary intake of iron and folate, infections (malaria, helminths),^36–38^ and poor supplementation during pregnancy (low coverage, inadequate counselling, insufficient supply and low adherence).^39–41^ Further investigations on diet, behaviour and supplementation bottlenecks are warranted, as well as on the association between income and anaemia which has not been widely documented in the region. In terms of overweight and obesity control, South Africa is the only country in the region currently implementing a sugar-sweetened beverage tax to reduce sugar intake and drive down the obesity epidemic^42^; similar policies have been successful at reducing the consumption of sugary drinks among high-volume intakers in Chile, Mexico, the UK and some US cities.^12^ Nevertheless, a combination of multiple strategies spanning across fiscal policies, regulatory policies on marketing, education, agriculture and food systems approaches will be needed to have a stronger impact^12^ and to curb the rising trends of overweight and obesity in SSA.

Our study has several strengths including the large span of countries and surveys covered since 2000, serving as a regional update on the nutritional status of both adult and adolescent women in SSA, as well as the novel analysis of within-urban disparities assessing capital city advantages or disadvantages. We were also faced with important limitations: the use of BMI-for-age Z score cut-offs to categorise underweight, overweight and obesity among adolescents may be a limitation in that they are not adjusted for the timing of adolescent growth spurts, and should be complemented with information on dietary intake.^43^ Furthermore, BMI as an anthropometric measure does not accurately reflect body fat composition nor the changes that occur with age; therefore, its use may result in misclassification errors.^44^ Nevertheless, large-scale household surveys are challenged by extensive data collection and do not include body composition variables; we, therefore, rely on BMI as the current standard by which underweight, overweight and obesity are defined using survey data. The multilevel trends were not longitudinal in design but rather a series of cross-sectional analyses of DHS surveys available by country; these differed in sample size. The regional estimates were unweighted in order to assign equal weights to all countries, and we assumed a linear relationship between the outcome and time. Assessment of scatterplots did not indicate any major departure from the linearity assumption. The lack of recent data remains an important limitation, with only 8 countries out of 35 in SSA having a survey in the last 5 years. Additionally, missing data ranged from 0% in Malawi 2004 to 13% in Lesotho 2004 for anaemia indicators, and 0.3% in Rwanda 2014 to 14% in Nigeria 1999 for underweight, overweight, obesity indicators; five countries did not include anaemia data in their most recent survey, and therefore, were not included in the respective anaemia analyses. Similarly, the surveys occurred at different years for each country, and therefore, the latest surveys cover a large period, which was restricted to 2010–2017. We are not comparing countries in terms of wealth or residence categories, since the context of poorest households or rural residents varies across countries; this calls for a cautious interpretation of our inequality analyses. Our sample was limited to women of reproductive age (15–49 years), as height and weight variables for men, younger adolescent girls (10–14 years) and older adult women (50+ years) are not collected.

In conclusion, our analysis of trends and inequalities in the nutritional status of adolescent girls and adult women in SSA suggests a rapidly rising overweight and obesity burden in parallel with persisting underweight and anaemia. The good news is that adolescent girls appear to be shielded from the rapid rise in overweight and obesity in the region; the bad is that adult women are bearing the brunt of the burden. Although anaemia has been declining, it remains a public health concern for all women, regardless of age, wealth, residence or education, further exacerbating the DBM. Our results also point to marked disparities in nutritional outcomes: although the prevalence of overweight and obesity remains largely concentrated among adult women, those living in urban areas and wealthier households, we suspect it will not be long before this burden transitions towards poor, rural communities, as is currently being witnessed in other regions of the world.^30^ Immediate equity-focused programmatic and policy responses are needed to address the triple burden of underweight, anaemia and overweight/obesity among women in SSA. Adolescent girls present a critical window of opportunity to act early on, and prevent the intergenerational cycle of malnutrition on pregnancy outcomes and child morbidity. Lastly, responses ought to have a double duty approach tackling both aspects of malnutrition, and be multifaceted to address socioeconomic and nutrition-sensitive factors playing a key role in the spread of this epidemic.
