# Supplementary material for: Trends and inequalities in the nutritional status of adolescent girls and adult women in sub-Saharan Africa since 2000: a cross-sectional series study
Source: BMJ Glob Health. 2020 Oct 8;5(10):e002948. doi: 10.1136/bmjgh-2020-002948 (PMC7545504; doi:10.1136/bmjgh-2020-002948)
Supplement: Supplementary data [file bmjgh-2020-002948supp001.pdf]

# Appendix

## Trends and inequalities in the nutritional status of adolescent girls and adult women in sub-Saharan Africa since 2000: a cross sectional series study.

Figures S1 A-E: Prevalence of underweight (A), anemia (B), anemia during pregnancy (C), overweight (D) and obesity (E) in Sub-Saharan Africa, using the last available survey (1994-2017)

A

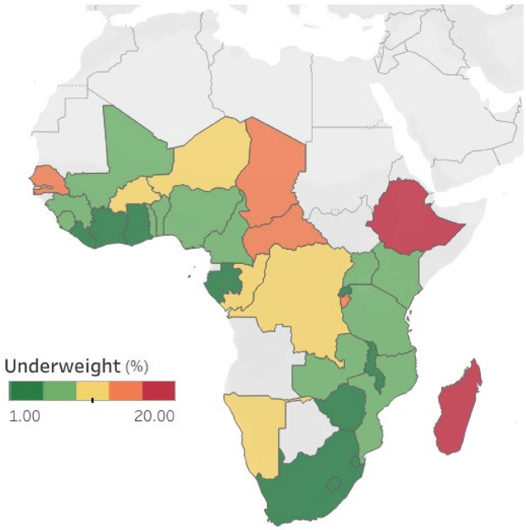


B

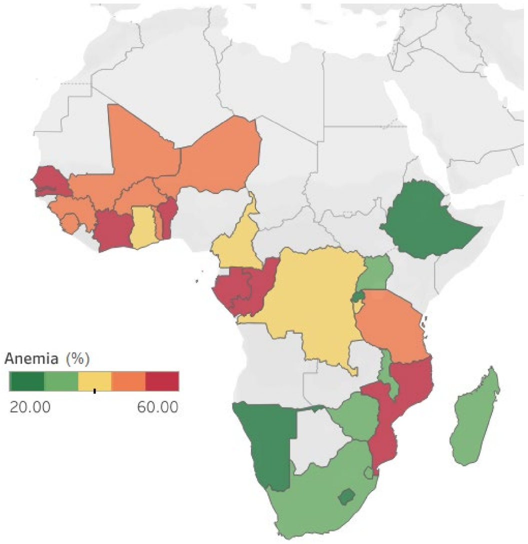


C

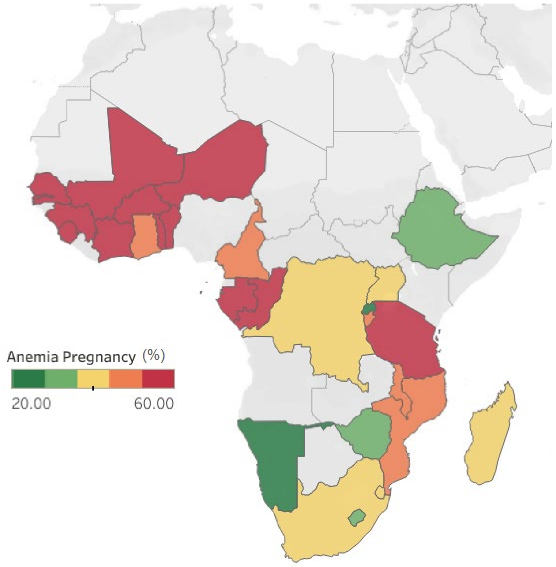

D

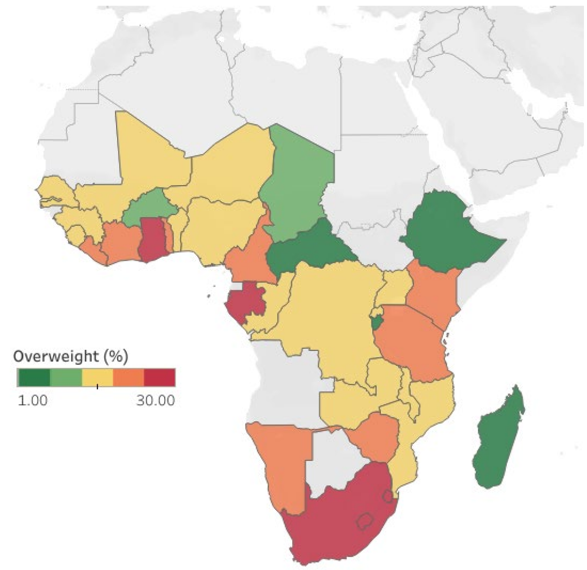


E

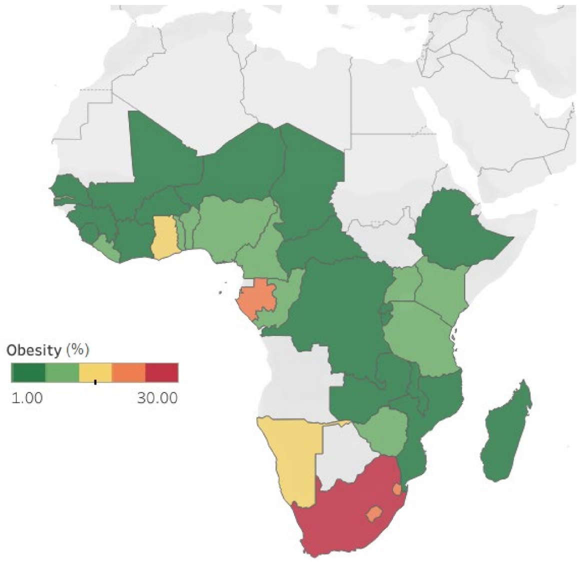

## Table S1: Prevalence of underweight by woman's age, wealth, residence and education using the latest surveys (2010-2017)

| Country, year | N | Underweight National % (95%CI) | Underweight by Woman's age % (95%CI) | | Underweight by Woman's Wealth % (95%CI) | | Underweight by Woman's Residence % (95%CI) | | | Underweight by Woman's Education % (95%CI) | | |
|---|---|---|---|---|---|---|---|---|---|---|---|---|
| | | | 15-19 years | 20-49 years | Poorest (Q1) | Richest (Q5) | Capital city | Other Urban | Rural | None | Primary | Secondary + |
| Benin 2017 | 6933 | 6.9 ( 6.2 , 7.6 ) | 1.3 ( 0.7 , 1.8 ) | 8.4 ( 7.6 , 9.3 ) | 10.8 ( 8.6 , 13.0 ) | 4.4 ( 3.4 , 5.4 ) | 3.4 ( 1.8 , 5.0 ) | 6.0 ( 4.9 , 7.0 ) | 7.8 ( 6.8 , 8.8 ) | 8.2 ( 7.1 , 9.2 ) | 5.4 ( 4.1 , 6.6 ) | 5.2 ( 4.2 , 6.3 ) |
| Burkina Faso 2010 | 7432 | 11.5 ( 10.6 , 12.5 ) | 3.3 ( 2.3 , 4.3 ) | 13.7 ( 12.5 , 14.9 ) | 19.0 ( 16.3 , 21.7 ) | 4.6 ( 3.4 , 5.8 ) | 4.5 ( 2.6 , 6.4 ) | 5.4 ( 4.1 , 6.7 ) | 14.1 ( 12.9 , 15.3 ) | 13.9 ( 12.7 , 15.1 ) | 5.2 ( 3.6 , 6.7 ) | 5.3 ( 3.6 , 7.0 ) |
| Burundi 2016 | 7714 | 14.5 ( 13.5 , 15.5 ) | 4.1 ( 3.1 , 5.1 ) | 17.8 ( 16.6 , 19.0 ) | 22.4 ( 20.0 , 24.7 ) | 8.2 ( 6.6 , 9.8 ) | 8.5 ( 5.5 , 11.6 ) | 8.1 ( 5.1 , 11.2 ) | 15.5 ( 14.4 , 16.5 ) | 21.4 ( 19.5 , 23.3 ) | 13.5 ( 12.1 , 14.9 ) | 6.9 ( 5.7 , 8.2 ) |
| Cameroon 2011 | 6991 | 4.9 ( 4.3 , 5.6 ) | 1.4 ( 0.7 , 2.1 ) | 6.0 ( 5.2 , 6.8 ) | 12.2 ( 9.4 , 15.0 ) | 2.0 ( 1.2 , 2.9 ) | 2.3 ( 1.2 , 3.3 ) | 4.2 ( 3.2 , 5.2 ) | 6.8 ( 5.7 , 8.0 ) | 13.7 ( 11.5 , 15.9 ) | 4.1 ( 3.2 , 5.1 ) | 2.1 ( 1.6 , 2.7 ) |
| Chad 2014 | 9426 | 14.4 ( 13.4 , 15.3 ) | 4.9 ( 3.9 , 6.0 ) | 17.2 ( 16.1 , 18.2 ) | 12.7 ( 10.8 , 14.5 ) | 9.5 ( 8.1 , 10.9 ) | 9.4 ( 7.2 , 11.6 ) | 11.0 ( 8.7 , 13.3 ) | 15.6 ( 14.5 , 16.7 ) | 19.2 ( 18.0 , 20.4 ) | 7.1 ( 5.7 , 8.4 ) | 6.2 ( 4.8 , 7.7 ) |
| Congo Brazzaville 2011 | 4927 | 9.6 ( 8.1 , 11.1 ) | 3.0 ( 1.2 , 4.8 ) | 11.4 ( 9.6 , 13.2 ) | 12.8 ( 10.8 , 14.8 ) | 5.0 ( 2.9 , 7.1 ) | 8.5 ( 5.6 , 11.5 ) | 9.2 ( 6.3 , 12.2 ) | 11.2 ( 9.8 , 12.5 ) | 12.6 ( 8.2 , 17.1 ) | 10.3 ( 8.4 , 12.2 ) | 9.1 ( 7.2 , 11.1 ) |
| Congo Democratic Republic 2013 | 7893 | 10.2 ( 9.2 , 11.2 ) | 2.6 ( 1.8 , 3.5 ) | 12.4 ( 11.2 , 13.7 ) | 15.6 ( 13.3 , 17.9 ) | 4.1 ( 3.0 , 5.2 ) | 4.0 ( 2.7 , 5.2 ) | 6.6 ( 5.2 , 8.0 ) | 12.9 ( 11.4 , 14.3 ) | 12.4 ( 10.2 , 14.5 ) | 12.6 ( 11.0 , 14.3 ) | 7.6 ( 6.2 , 9.0 ) |
| Cote d'Ivoire 2011 | 4195 | 4.6 ( 3.8 , 5.4 ) | 1.6 ( 0.5 , 2.8 ) | 5.4 ( 4.4 , 6.3 ) | 5.7 ( 4.0 , 7.5 ) | 4.1 ( 2.6 , 5.7 ) | 4.4 ( 2.4 , 6.3 ) | 4.0 ( 2.6 , 5.3 ) | 5.0 ( 3.9 , 6.1 ) | 4.5 ( 3.5 , 5.5 ) | 4.8 ( 3.1 , 6.5 ) | 4.4 ( 2.8 , 6.0 ) |
| Ethiopia 2016 | 13434 | 17.0 ( 16.1 , 18.0 ) | 5.1 ( 4.0 , 6.2 ) | 20.5 ( 19.4 , 21.6 ) | 23.2 ( 20.4 , 25.9 ) | 11.3 ( 9.8 , 12.8 ) | 9.2 ( 7.6 , 10.9 ) | 11.3 ( 9.4 , 13.3 ) | 18.9 ( 17.8 , 20.0 ) | 21.7 ( 20.3 , 23.1 ) | 13.5 ( 12.0 , 14.9 ) | 11.5 ( 9.7 , 13.3 ) |
| Gabon 2012 | 4841 | 3.8 ( 3.0 , 4.6 ) | 1.3 ( 0.3 , 2.2 ) | 4.5 ( 3.5 , 5.5 ) | 5.5 ( 4.2 , 6.7 ) | 3.4 ( 1.6 , 5.2 ) | 3.2 ( 1.9 , 4.5 ) | 4.4 ( 3.4 , 5.4 ) | 5.2 ( 3.9 , 6.6 ) | 1.3 ( 0.1 , 2.6 ) | 3.0 ( 2.0 , 4.0 ) | 4.2 ( 3.1 , 5.3 ) |
| Gambia 2013 | 4061 | 11.2 ( 9.8 , 12.6 ) | 4.1 ( 2.5 , 5.7 ) | 13.4 ( 11.7 , 15.2 ) | 12.5 ( 9.8 , 15.1 ) | 7.0 ( 4.6 , 9.5 ) | 9.0 ( 6.5 , 11.5 ) | 9.8 ( 7.7 , 11.9 ) | 13.0 ( 11.3 , 14.8 ) | 12.6 ( 10.3 , 14.9 ) | 7.9 ( 5.4 , 10.4 ) | 10.7 ( 8.7 , 12.7 ) |
| Ghana 2014 | 4314 | 3.8 ( 3.2 , 4.5 ) | 1.6 ( 0.7 , 2.5 ) | 4.3 ( 3.5 , 5.1 ) | 6.7 ( 5.2 , 8.2 ) | 2.2 ( 1.1 , 3.3 ) | 2.6 ( 1.1 , 4.0 ) | 3.6 ( 2.6 , 4.7 ) | 4.5 ( 3.5 , 5.6 ) | 5.7 ( 4.3 , 7.1 ) | 4.2 ( 2.5 , 5.8 ) | 3.2 ( 2.4 , 4.0 ) |
| Guinea 2012 | 4131 | 8.3 ( 7.1 , 9.4 ) | 2.3 ( 1.2 , 3.5 ) | 10.1 ( 8.7 , 11.6 ) | 13.9 ( 11.2 , 16.6 ) | 5.3 ( 3.4 , 7.1 ) | 5.4 ( 2.8 , 8.1 ) | 4.8 ( 3.3 , 6.2 ) | 10.0 ( 8.4 , 11.6 ) | 10.1 ( 8.7 , 11.5 ) | 5.4 ( 3.4 , 7.5 ) | 4.4 ( 2.8 , 6.0 ) |
| Kenya 2014 | 13213 | 6.2 ( 5.7 , 6.8 ) | 2.5 ( 1.8 , 3.1 ) | 7.1 ( 6.5 , 7.7 ) | 16.4 ( 14.5 , 18.4 ) | 2.8 ( 2.0 , 3.6 ) | 2.1 ( 0.8 , 3.5 ) | 4.5 ( 3.6 , 5.3 ) | 7.8 ( 7.1 , 8.6 ) | 22.8 ( 19.8 , 25.9 ) | 5.8 ( 5.2 , 6.5 ) | 4.2 ( 3.5 , 4.9 ) |
| Lesotho 2014 | 3193 | 2.5 ( 1.9 , 3.2 ) | 1.0 ( -0.3 , 2.3 ) | 3.0 ( 2.1 , 3.8 ) | 3.3 ( 1.7 , 4.9 ) | 2.7 ( 1.2 , 4.3 ) | 3.8 ( 1.3 , 6.3 ) | 2.1 ( 0.8 , 3.4 ) | 2.3 ( 1.7 , 3.0 ) | 2.7 ( -1.5 , 7.0 ) | 2.7 ( 1.7 , 3.6 ) | 2.5 ( 1.5 , 3.4 ) |
| Liberia 2013 | 4087 | 4.4 ( 3.6 , 5.2 ) | 1.4 ( 0.3 , 2.5 ) | 5.3 ( 4.3 , 6.2 ) | 7.2 ( 5.4 , 9.0 ) | 2.9 ( 1.3 , 4.6 ) | 2.8 ( 1.4 , 4.2 ) | 4.6 ( 3.2 , 6.1 ) | 5.9 ( 4.7 , 7.0 ) | 6.7 ( 5.1 , 8.3 ) | 4.8 ( 3.0 , 6.6 ) | 2.1 ( 1.1 , 3.2 ) |
| Malawi 2015 | 7275 | 4.6 ( 4.0 , 5.2 ) | 1.1 ( 0.5 , 1.7 ) | 5.6 ( 4.9 , 6.3 ) | 6.6 ( 5.1 , 8.1 ) | 3.4 ( 2.4 , 4.5 ) | 4.9 ( 1.3 , 8.4 ) | 3.3 ( 2.1 , 4.5 ) | 4.8 ( 4.2 , 5.4 ) | 6.7 ( 4.8 , 8.6 ) | 4.5 ( 3.8 , 5.2 ) | 4.0 ( 2.8 , 5.2 ) |
| Mali 2012 | 4509 | 8.5 ( 7.5 , 9.6 ) | 2.6 ( 1.4 , 3.7 ) | 9.9 ( 8.6 , 11.1 ) | 10.3 ( 8.0 , 12.6 ) | 4.6 ( 3.3 , 5.8 ) | 4.6 ( 3.0 , 6.1 ) | 6.0 ( 3.2 , 8.8 ) | 9.7 ( 8.4 , 11.0 ) | 9.8 ( 8.5 , 11.1 ) | 6.2 ( 4.0 , 8.4 ) | 4.1 ( 2.6 , 5.6 ) |
| Mozambique 2011 | 11877 | 5.7 ( 5.1 , 6.2 ) | 1.6 ( 1.0 , 2.2 ) | 6.8 ( 6.1 , 7.5 ) | 9.3 ( 7.6 , 11.0 ) | 3.2 ( 2.5 , 3.9 ) | 3.2 ( 2.3 , 4.1 ) | 4.3 ( 3.5 , 5.1 ) | 6.6 ( 5.8 , 7.4 ) | 8.4 ( 7.1 , 9.7 ) | 4.8 ( 4.1 , 5.5 ) | 3.5 ( 2.7 , 4.4 ) |
| Namibia 2013 | 4008 | 9.4 ( 8.3 , 10.6 ) | 5.1 ( 3.5 , 6.6 ) | 10.6 ( 9.2 , 11.9 ) | 17.2 ( 13.7 , 20.8 ) | 4.4 ( 2.4 , 6.4 ) | 6.3 ( 2.9 , 9.7 ) | 7.8 ( 6.3 , 9.3 ) | 12.0 ( 10.4 , 13.7 ) | 13.1 ( 8.3 , 18.0 ) | 13.0 ( 10.2 , 15.8 ) | 8.2 ( 6.9 , 9.5 ) |
| Niger 2012 | 4243 | 11.3 ( 10.1 , 12.6 ) | 6.3 ( 4.3 , 8.4 ) | 12.4 ( 10.9 , 13.8 ) | 15.6 ( 12.5 , 18.8 ) | 6.2 ( 4.7 , 7.6 ) | 6.3 ( 3.9 , 8.6 ) | 7.3 ( 5.0 , 9.6 ) | 12.3 ( 10.9 , 13.8 ) | 12.3 ( 10.8 , 13.7 ) | 7.8 ( 5.0 , 10.7 ) | 7.1 ( 4.8 , 9.4 ) |
| Nigeria 2013 | 33063 | 7.4 ( 7.0 , 7.9 ) | 4.1 ( 3.4 , 4.8 ) | 8.3 ( 7.8 , 8.8 ) | 12.9 ( 11.5 , 14.3 ) | 3.3 ( 2.8 , 3.8 ) | 4.9 ( 3.4 , 6.5 ) | 5.8 ( 5.1 , 6.4 ) | 8.8 ( 8.1 , 9.4 ) | 11.2 ( 10.3 , 12.1 ) | 7.2 ( 6.3 , 8.0 ) | 4.6 ( 4.1 , 5.0 ) |
| Rwanda 2014 | 6108 | 4.4 ( 3.9 , 5.0 ) | 1.2 ( 0.6 , 1.9 ) | 5.3 ( 4.7 , 6.0 ) | 7.8 ( 6.2 , 9.5 ) | 3.2 ( 2.4 , 4.1 ) | 4.3 ( 2.8 , 5.7 ) | 3.4 ( 2.1 , 4.7 ) | 4.6 ( 4.0 , 5.2 ) | 7.7 ( 5.7 , 9.8 ) | 4.2 ( 3.5 , 4.9 ) | 3.4 ( 2.5 , 4.3 ) |
| Senegal 2010 | 5128 | 15.8 ( 14.2 , 17.3 ) | 8.2 ( 6.3 , 10.2 ) | 18.1 ( 16.2 , 20.0 ) | 19.7 ( 16.9 , 22.5 ) | 10.9 ( 8.2 , 13.7 ) | 12.8 ( 8.7 , 16.8 ) | 13.1 ( 10.9 , 15.2 ) | 18.6 ( 16.5 , 20.7 ) | 18.2 ( 16.3 , 20.0 ) | 13.8 ( 10.6 , 16.9 ) | 11.6 ( 8.7 , 14.6 ) |
| Sierra Leone 2013 | 7162 | 6.2 ( 5.5 , 7.0 ) | 2.6 ( 1.6 , 3.7 ) | 7.3 ( 6.3 , 8.2 ) | 8.3 ( 6.4 , 10.3 ) | 3.7 ( 2.5 , 4.9 ) | 3.8 ( 2.0 , 5.5 ) | 4.7 ( 3.5 , 5.9 ) | 7.4 ( 6.3 , 8.4 ) | 7.3 ( 6.3 , 8.3 ) | 6.1 ( 4.1 , 8.1 ) | 4.3 ( 3.3 , 5.3 ) |
| South Africa 2016 | 3210 | 2.1 ( 1.5 , 2.7 ) | 0.3 ( 0.0 , 0.7 ) | 2.4 ( 1.7 , 3.1 ) | 1.7 ( 0.8 , 2.6 ) | 1.7 ( 0.0 , 3.4 ) | 2.2 ( 1.0 , 3.4 ) | 1.5 ( 0.7 , 2.4 ) | 2.3 ( 1.5 , 3.2 ) | 1.0 ( -0.7 , 2.8 ) | 2.7 ( 0.0 , 5.5 ) | 2.0 ( 1.4 , 2.6 ) |
| Tanzania 2015 | 11735 | 6.0 ( 5.4 , 6.5 ) | 2.4 ( 1.6 , 3.1 ) | 7.0 ( 6.4 , 7.7 ) | 9.3 ( 7.7 , 10.9 ) | 3.6 ( 2.8 , 4.4 ) | 4.5 ( 3.1 , 6.0 ) | 4.6 ( 3.6 , 5.6 ) | 6.8 ( 6.1 , 7.5 ) | 8.5 ( 6.8 , 10.3 ) | 5.7 ( 5.1 , 6.4 ) | 5.1 ( 4.1 , 6.2 ) |
| Togo 2013 | 4326 | 4.8 ( 4.1 , 5.5 ) | 0.9 ( 0.4 , 1.5 ) | 5.8 ( 4.9 , 6.6 ) | 8.9 ( 7.0 , 10.7 ) | 2.7 ( 1.7 , 3.7 ) | 2.9 ( 1.7 , 4.1 ) | 2.9 ( 1.4 , 4.5 ) | 6.5 ( 5.5 , 7.5 ) | 7.0 ( 5.6 , 8.3 ) | 4.0 ( 2.9 , 5.2 ) | 3.8 ( 2.7 , 4.8 ) |
| Uganda 2016 | 5240 | 5.9 ( 5.1 , 6.6 ) | 0.9 ( 0.4 , 1.3 ) | 7.4 ( 6.5 , 8.4 ) | 12.1 ( 9.9 , 14.2 ) | 2.6 ( 1.5 , 3.7 ) | 3.1 ( 1.3 , 4.9 ) | 5.0 ( 3.2 , 6.7 ) | 6.3 ( 5.5 , 7.2 ) | 11.6 ( 8.5 , 14.7 ) | 6.3 ( 5.3 , 7.3 ) | 3.4 ( 2.3 , 4.5 ) |
| Zambia 2013 | 14510 | 6.9 ( 6.4 , 7.5 ) | 1.7 ( 1.2 , 2.2 ) | 8.5 ( 7.8 , 9.1 ) | 11.9 ( 10.4 , 13.4 ) | 4.4 ( 3.4 , 5.4 ) | 4.9 ( 3.8 , 6.1 ) | 5.4 ( 4.5 , 6.3 ) | 8.5 ( 7.7 , 9.3 ) | 11.3 ( 9.2 , 13.4 ) | 8.1 ( 7.4 , 8.9 ) | 4.9 ( 4.2 , 5.6 ) |
| Zimbabwe 2010 | 7931 | 4.5 ( 4.0 , 5.0 ) | 1.6 ( 0.9 , 2.2 ) | 5.3 ( 4.7 , 6.0 ) | 7.3 ( 5.9 , 8.8 ) | 2.8 ( 1.9 , 3.6 ) | 3.4 ( 2.2 , 4.7 ) | 3.2 ( 2.3 , 4.1 ) | 5.3 ( 4.5 , 6.0 ) | 4.6 ( 1.7 , 7.6 ) | 4.9 ( 4.0 , 5.9 ) | 4.3 ( 3.7 , 5.0 ) |

Table S2: Prevalence of anemia by woman's age, wealth, residence and education using the latest surveys (2010-2017)

| Country, year | N | Anemia National % (95%CI) | Anemia by Woman's age % (95%CI) | | Anemia by Woman's Wealth % (95%CI) | | Anemia by Woman's Residence % (95%CI) | | | Anemia by Woman's Education % (95%CI) | | |
|---|---|---|---|---|---|---|---|---|---|---|---|---|
| | | | 15-19 years | 20-49 years | Poorest (Q1) | Richest (Q5) | Capital city | Other Urban | Rural | None | Primary | Secondary + |
| Benin 2017 | 8011 | 57.7 ( 56.2 , 59.3 ) | 57.4 ( 54.5 , 60.3 ) | 57.8 ( 56.2 , 59.4 ) | 63.1 ( 59.7 , 66.5 ) | 54.8 ( 52.0 , 57.6 ) | 60.7 ( 56.5 , 64.8 ) | 57.0 ( 54.6 , 59.4 ) | 57.9 ( 55.8 , 60.0 ) | 59.3 ( 57.3 , 61.2 ) | 58.6 ( 55.7 , 61.4 ) | 53.7 ( 51.2 , 56.2 ) |
| Burkina Faso 2010 | 8376 | 48.8 ( 47.3 , 50.4 ) | 47.9 ( 45.2 , 50.7 ) | 49.1 ( 47.4 , 50.7 ) | 54.6 ( 51.4 , 57.9 ) | 42.3 ( 39.0 , 45.6 ) | 42.0 ( 36.7 , 47.3 ) | 43.0 ( 39.6 , 46.5 ) | 51.1 ( 49.3 , 52.9 ) | 51.1 ( 49.4 , 52.8 ) | 43.2 ( 39.8 , 46.5 ) | 41.1 ( 36.5 , 45.7 ) |
| Burundi 2016 | 8539 | 39.3 ( 37.7 , 40.9 ) | 35.9 ( 33.2 , 38.6 ) | 40.2 ( 38.5 , 41.9 ) | 50.3 ( 47.4 , 53.3 ) | 25.2 ( 22.8 , 27.6 ) | 21.2 ( 17.8 , 24.5 ) | 23.2 ( 19.9 , 26.4 ) | 41.9 ( 40.2 , 43.6 ) | 45.8 ( 43.7 , 48.0 ) | 41.1 ( 38.8 , 43.5 ) | 27.0 ( 24.7 , 29.3 ) |
| Cameroon 2011 | 7819 | 39.5 ( 37.9 , 41.1 ) | 39.7 ( 36.8 , 42.5 ) | 39.5 ( 37.8 , 41.1 ) | 36.0 ( 32.0 , 40.0 ) | 42.3 ( 39.4 , 45.2 ) | 44.8 ( 41.6 , 48.0 ) | 38.9 ( 36.5 , 41.4 ) | 37.3 ( 34.8 , 39.7 ) | 39.4 ( 35.2 , 43.6 ) | 36.9 ( 34.7 , 39.0 ) | 41.5 ( 39.6 , 43.5 ) |
| Chad 2014 | | | | | | | | | | | | |
| Congo Brazzaville 2011 | 5562 | 54.2 ( 52.1 , 56.3 ) | 55.1 ( 50.0 , 60.2 ) | 53.9 ( 51.4 , 56.5 ) | 48.3 ( 45.7 , 51.0 ) | 53.9 ( 48.4 , 59.5 ) | 53.5 ( 48.5 , 58.5 ) | 59.0 ( 55.9 , 62.1 ) | 49.8 ( 47.6 , 52.1 ) | 52.1 ( 45.8 , 58.3 ) | 53.2 ( 49.6 , 56.7 ) | 54.7 ( 51.7 , 57.7 ) |
| Congo Democratic Republic 2013 | 9328 | 38.4 ( 36.1 , 40.6 ) | 40.1 ( 36.6 , 43.7 ) | 37.9 ( 35.5 , 40.2 ) | 37.8 ( 34.5 , 41.0 ) | 41.3 ( 37.5 , 45.2 ) | 46.7 ( 43.6 , 49.8 ) | 37.9 ( 34.2 , 41.6 ) | 37.1 ( 34.0 , 40.2 ) | 32.9 ( 29.5 , 36.3 ) | 38.2 ( 35.3 , 41.1 ) | 40.4 ( 37.8 , 43.0 ) |
| Cote d'Ivoire 2011 | 4678 | 53.7 ( 51.4 , 56.1 ) | 53.9 ( 49.4 , 58.3 ) | 53.7 ( 51.3 , 56.2 ) | 54.8 ( 49.5 , 60.1 ) | 49.8 ( 43.9 , 55.8 ) | 53.1 ( 46.2 , 59.9 ) | 52.6 ( 48.7 , 56.6 ) | 54.7 ( 51.9 , 57.4 ) | 55.3 ( 52.7 , 57.9 ) | 54.3 ( 50.6 , 57.9 ) | 48.8 ( 43.7 , 54.0 ) |
| Ethiopia 2016 | 14489 | 23.6 ( 22.0 , 25.3 ) | 19.9 ( 17.8 , 22.1 ) | 24.6 ( 22.8 , 26.5 ) | 34.3 ( 29.5 , 39.0 ) | 17.4 ( 15.0 , 19.8 ) | 16.0 ( 13.3 , 18.6 ) | 17.4 ( 13.7 , 21.0 ) | 25.4 ( 23.5 , 27.3 ) | 27.8 ( 25.4 , 30.1 ) | 21.7 ( 19.7 , 23.6 ) | 15.8 ( 12.9 , 18.6 ) |
| Gabon 2012 | 5484 | 60.6 ( 58.3 , 62.9 ) | 63.5 ( 59.4 , 67.7 ) | 59.8 ( 57.2 , 62.3 ) | 54.1 ( 50.9 , 57.3 ) | 58.6 ( 52.1 , 65.1 ) | 61.5 ( 57.8 , 65.2 ) | 60.3 ( 57.8 , 62.8 ) | 56.9 ( 53.6 , 60.2 ) | 47.6 ( 38.0 , 57.2 ) | 58.8 ( 54.6 , 63.0 ) | 62.0 ( 59.6 , 64.5 ) |
| Gambia 2013 | 4478 | 60.3 ( 57.6 , 62.9 ) | 58.2 ( 53.9 , 62.5 ) | 60.9 ( 58.1 , 63.7 ) | 67.3 ( 63.0 , 71.5 ) | 49.0 ( 44.1 , 53.9 ) | 52.7 ( 46.7 , 58.6 ) | 53.3 ( 49.1 , 57.5 ) | 68.4 ( 65.5 , 71.2 ) | 64.6 ( 61.8 , 67.5 ) | 62.8 ( 57.8 , 67.7 ) | 54.0 ( 50.2 , 57.9 ) |
| Ghana 2014 | 4704 | 42.4 ( 40.5 , 44.2 ) | 47.7 ( 43.8 , 51.6 ) | 41.2 ( 39.2 , 43.3 ) | 43.6 ( 39.6 , 47.6 ) | 37.7 ( 33.2 , 42.2 ) | 43.8 ( 38.0 , 49.6 ) | 40.8 ( 37.9 , 43.6 ) | 43.0 ( 40.5 , 45.5 ) | 45.5 ( 42.1 , 48.9 ) | 44.6 ( 40.4 , 48.8 ) | 40.7 ( 38.5 , 42.9 ) |
| Guinea 2012 | 4691 | 49.1 ( 47.1 , 51.2 ) | 47.1 ( 43.6 , 50.6 ) | 49.8 ( 47.4 , 52.2 ) | 55.8 ( 51.4 , 60.2 ) | 42.6 ( 38.4 , 46.9 ) | 42.6 ( 37.3 , 47.9 ) | 44.6 ( 41.1 , 48.1 ) | 52.2 ( 49.5 , 54.8 ) | 51.6 ( 49.3 , 54.0 ) | 48.6 ( 44.4 , 52.8 ) | 40.8 ( 37.2 , 44.3 ) |
| Kenya 2014 | | | | | | | | | | | | |
| Lesotho 2014 | 3349 | 27.3 ( 25.3 , 29.2 ) | 24.1 ( 19.7 , 28.5 ) | 28.2 ( 26.2 , 30.2 ) | 22.8 ( 18.8 , 26.7 ) | 28.9 ( 24.7 , 33.0 ) | 38.6 ( 31.4 , 45.8 ) | 26.3 ( 23.3 , 29.2 ) | 24.8 ( 22.8 , 26.7 ) | 31.2 ( 11.2 , 51.3 ) | 25.6 ( 22.9 , 28.2 ) | 28.3 ( 25.7 , 30.9 ) |
| Liberia 2013 | | | | | | | | | | | | |
| Malawi 2015 | 7970 | 32.7 ( 31.3 , 34.2 ) | 35.3 ( 32.5 , 38.2 ) | 32.0 ( 30.5 , 33.6 ) | 32.0 ( 28.9 , 35.0 ) | 34.3 ( 31.3 , 37.4 ) | 39.5 ( 32.8 , 46.1 ) | 33.4 ( 29.6 , 37.1 ) | 32.1 ( 30.5 , 33.7 ) | 34.5 ( 30.8 , 38.2 ) | 31.9 ( 30.1 , 33.7 ) | 33.8 ( 31.1 , 36.5 ) |
| Mali 2012 | 5160 | 51.4 ( 49.6 , 53.2 ) | 50.8 ( 46.7 , 54.8 ) | 51.6 ( 49.6 , 53.5 ) | 58.8 ( 54.8 , 62.7 ) | 42.1 ( 39.3 , 44.9 ) | 46.3 ( 42.7 , 49.9 ) | 42.1 ( 38.3 , 45.8 ) | 53.7 ( 51.5 , 55.9 ) | 54.0 ( 52.0 , 56.1 ) | 47.2 ( 41.3 , 53.0 ) | 41.0 ( 36.6 , 45.4 ) |
| Mozambique 2011 | 13537 | 54.0 ( 52.3 , 55.6 ) | 54.9 ( 52.4 , 57.4 ) | 53.7 ( 52.0 , 55.5 ) | 62.5 ( 58.7 , 66.2 ) | 50.1 ( 47.7 , 52.6 ) | 48.7 ( 45.4 , 52.0 ) | 52.5 ( 49.5 , 55.4 ) | 55.1 ( 53.0 , 57.3 ) | 57.7 ( 55.1 , 60.3 ) | 53.0 ( 51.1 , 54.9 ) | 50.1 ( 47.4 , 52.7 ) |
| Namibia 2013 | 4327 | 20.7 ( 19.2 , 22.1 ) | 19.1 ( 16.1 , 22.1 ) | 21.1 ( 19.4 , 22.7 ) | 24.0 ( 20.6 , 27.4 ) | 17.9 ( 14.7 , 21.2 ) | 15.8 ( 12.3 , 19.2 ) | 21.3 ( 18.9 , 23.6 ) | 22.4 ( 20.4 , 24.4 ) | 26.8 ( 20.9 , 32.7 ) | 24.0 ( 21.0 , 27.1 ) | 19.4 ( 17.8 , 20.9 ) |
| Niger 2012 | 5050 | 45.8 ( 43.5 , 48.1 ) | 46.0 ( 41.5 , 50.5 ) | 45.8 ( 43.4 , 48.1 ) | 52.4 ( 47.7 , 57.1 ) | 40.1 ( 36.3 , 43.9 ) | 47.4 ( 42.4 , 52.3 ) | 38.3 ( 32.0 , 44.5 ) | 46.5 ( 43.9 , 49.1 ) | 46.7 ( 44.3 , 49.2 ) | 42.8 ( 38.2 , 47.5 ) | 40.8 ( 35.8 , 45.8 ) |
| Nigeria 2013 | | | | | | | | | | | | |
| Rwanda 2014 | 6692 | 19.2 ( 18.0 , 20.5 ) | 18.8 ( 16.4 , 21.1 ) | 19.4 ( 18.0 , 20.7 ) | 24.8 ( 22.1 , 27.5 ) | 16.6 ( 14.5 , 18.7 ) | 14.8 ( 11.5 , 18.0 ) | 18.2 ( 15.7 , 20.7 ) | 19.9 ( 18.5 , 21.4 ) | 22.5 ( 19.3 , 25.6 ) | 19.1 ( 17.6 , 20.6 ) | 17.9 ( 15.9 , 19.9 ) |
| Senegal 2017 | 8013 | 54.1 ( 52.2 , 56.0 ) | 57.2 ( 53.8 , 60.6 ) | 53.2 ( 51.2 , 55.2 ) | 60.6 ( 57.5 , 63.6 ) | 50.9 ( 46.4 , 55.3 ) | 52.1 ( 47.0 , 57.3 ) | 48.5 ( 46.1 , 50.9 ) | 57.6 ( 55.2 , 60.0 ) | 57.8 ( 55.6 , 60.0 ) | 53.3 ( 50.0 , 56.7 ) | 49.2 ( 45.9 , 52.5 ) |
| Sierra Leone 2013 | 7848 | 44.8 ( 42.4 , 47.2 ) | 49.5 ( 46.3 , 52.6 ) | 43.5 ( 40.9 , 46.1 ) | 50.6 ( 47.1 , 54.2 ) | 34.1 ( 28.0 , 40.1 ) | 29.4 ( 20.7 , 38.1 ) | 43.0 ( 41.0 , 44.9 ) | 49.2 ( 46.9 , 51.5 ) | 47.0 ( 44.6 , 49.3 ) | 47.4 ( 43.8 , 50.9 ) | 39.4 ( 34.7 , 44.2 ) |
| South Africa 2016 | 2974 | 33.2 ( 30.5 , 35.8 ) | 33.9 ( 29.0 , 38.8 ) | 33.0 ( 30.0 , 36.1 ) | 29.6 ( 25.3 , 33.9 ) | 31.9 ( 24.7 , 39.0 ) | 33.3 ( 28.4 , 38.3 ) | 31.8 ( 28.0 , 35.5 ) | 33.9 ( 29.6 , 38.2 ) | 25.5 ( 10.6 , 40.3 ) | 36.1 ( 29.2 , 43.1 ) | 33.0 ( 30.4 , 35.6 ) |
| Tanzania 2015 | 13102 | 44.8 ( 43.4 , 46.3 ) | 47.3 ( 45.0 , 49.6 ) | 44.1 ( 42.6 , 45.7 ) | 48.5 ( 45.3 , 51.7 ) | 43.7 ( 41.0 , 46.4 ) | 53.1 ( 48.1 , 58.1 ) | 40.6 ( 37.7 , 43.4 ) | 45.0 ( 43.3 , 46.8 ) | 51.2 ( 48.5 , 54.0 ) | 44.5 ( 42.7 , 46.3 ) | 41.8 ( 39.7 , 43.8 ) |
| Togo 2013 | 4802 | 48.1 ( 46.3 , 49.9 ) | 54.7 ( 51.1 , 58.4 ) | 46.6 ( 44.6 , 48.5 ) | 41.2 ( 38.0 , 44.4 ) | 51.5 ( 47.5 , 55.5 ) | 55.8 ( 52.3 , 59.4 ) | 42.6 ( 37.0 , 48.2 ) | 45.3 ( 43.1 , 47.5 ) | 47.0 ( 43.7 , 50.3 ) | 47.5 ( 44.6 , 50.4 ) | 49.7 ( 46.7 , 52.7 ) |
| Uganda 2016 | 6031 | 31.7 ( 30.1 , 33.2 ) | 32.9 ( 30.0 , 35.8 ) | 31.3 ( 29.6 , 33.0 ) | 40.6 ( 37.0 , 44.2 ) | 25.2 ( 22.7 , 27.6 ) | 25.2 ( 20.4 , 30.0 ) | 27.9 ( 24.5 , 31.4 ) | 33.2 ( 31.4 , 35.0 ) | 36.6 ( 32.3 , 41.0 ) | 31.7 ( 29.8 , 33.6 ) | 30.2 ( 27.8 , 32.6 ) |
| Zambia 2013 | | | | | | | | | | | | |
| Zimbabwe 2010 | 8155 | 28.2 ( 26.9 , 29.5 ) | 25.7 ( 23.5 , 27.9 ) | 28.9 ( 27.5 , 30.3 ) | 28.0 ( 24.7 , 31.3 ) | 28.9 ( 26.6 , 31.3 ) | 27.2 ( 24.5 , 29.9 ) | 33.9 ( 31.2 , 36.6 ) | 26.9 ( 25.2 , 28.5 ) | 29.8 ( 22.3 , 37.3 ) | 27.8 ( 25.4 , 30.2 ) | 28.3 ( 27.0 , 29.7 ) |

Table S3: Prevalence of anemia during pregnancy by woman's age, wealth, residence and education using the latest surveys (2010-2017)

| Country, year | N | Anemia Pregnancy National % (95%CI) | Anemia Pregnancy by Woman's age % (95%CI) | | Anemia Pregnancy by Woman's Wealth % (95%CI) | | Anemia Pregnancy by Woman's Residence % (95%CI) | | | Anemia Pregnancy by Woman's Education % (95%CI) | | |
|---|---|---|---|---|---|---|---|---|---|---|---|---|
| | | | 15-19 years | 20-49 years | Poorest (Q1) | Richest (Q5) | Capital city | Other Urban | Rural | None | Primary | Secondary + |
| Benin 2017 | 878 | 68.4 ( 64.7 , 72.0 ) | 66.7 ( 57.0 , 76.5 ) | 68.6 ( 64.8 , 72.4 ) | 73.5 ( 66.2 , 80.9 ) | 58.2 ( 49.1 , 67.2 ) | 74.5 ( 61.4 , 87.5 ) | 68.3 ( 61.8 , 74.8 ) | 68.1 ( 63.5 , 72.6 ) | 69.8 ( 65.4 , 74.2 ) | 66.3 ( 58.8 , 73.8 ) | 66.0 ( 58.1 , 73.8 ) |
| Burkina Faso 2010 | 820 | 58.2 ( 54.5 , 61.8 ) | 77.3 ( 68.2 , 86.4 ) | 55.7 ( 51.7 , 59.7 ) | 59.9 ( 51.7 , 68.0 ) | 48.3 ( 39.4 , 57.2 ) | 52.9 ( 37.9 , 67.9 ) | 52.5 ( 44.1 , 61.0 ) | 59.4 ( 55.3 , 63.4 ) | 60.5 ( 56.4 , 64.5 ) | 51.4 ( 40.6 , 62.2 ) | 43.1 ( 28.2 , 58.0 ) |
| Burundi 2016 | 664 | 46.8 ( 42.4 , 51.1 ) | 48.8 ( 32.2 , 65.4 ) | 46.6 ( 42.1 , 51.1 ) | 48.8 ( 40.3 , 57.4 ) | 31.9 ( 22.1 , 41.8 ) | 24.9 ( 6.2 , 43.7 ) | 38.2 ( 26.0 , 50.4 ) | 49.0 ( 44.5 , 53.4 ) | 50.1 ( 43.9 , 56.4 ) | 47.7 ( 41.5 , 54.0 ) | 35.0 ( 24.5 , 45.4 ) |
| Cameroon 2011 | 752 | 49.9 ( 45.5 , 54.3 ) | 54.2 ( 43.3 , 65.2 ) | 49.0 ( 44.3 , 53.6 ) | 44.5 ( 35.2 , 53.8 ) | 51.0 ( 42.0 , 60.0 ) | 53.3 ( 42.6 , 64.1 ) | 56.6 ( 48.2 , 65.1 ) | 45.6 ( 39.9 , 51.3 ) | 47.1 ( 37.8 , 56.4 ) | 48.6 ( 41.9 , 55.4 ) | 53.1 ( 46.7 , 59.6 ) |
| Chad 2014 | | | | | | | | | | | | |
| Congo Brazzaville 2011 | 582 | 58.4 ( 51.6 , 65.2 ) | 60.3 ( 45.3 , 75.3 ) | 57.9 ( 50.2 , 65.6 ) | 62.4 ( 56.0 , 68.9 ) | 39.3 ( 19.9 , 58.8 ) | 60.1 ( 43.0 , 77.2 ) | 56.6 ( 42.4 , 70.8 ) | 59.0 ( 53.1 , 64.9 ) | 63.9 ( 44.1 , 83.7 ) | 57.7 ( 48.6 , 66.8 ) | 58.2 ( 49.8 , 66.6 ) |
| Congo Democratic Republic 2013 | 1203 | 43.4 ( 39.3 , 47.5 ) | 46.0 ( 36.3 , 55.7 ) | 42.9 ( 38.3 , 47.5 ) | 44.1 ( 37.0 , 51.3 ) | 57.0 ( 45.6 , 68.5 ) | 62.3 ( 45.4 , 79.1 ) | 48.8 ( 41.5 , 56.2 ) | 40.0 ( 35.0 , 45.0 ) | 32.9 ( 24.9 , 40.8 ) | 43.2 ( 36.5 , 49.9 ) | 47.6 ( 41.2 , 53.9 ) |
| Cote d'Ivoire 2011 | 481 | 63.6 ( 58.6 , 68.6 ) | 64.8 ( 51.2 , 78.5 ) | 63.4 ( 57.9 , 68.8 ) | 64.5 ( 52.0 , 77.1 ) | 56.2 ( 40.4 , 72.0 ) | 74.6 ( 59.6 , 89.5 ) | 59.2 ( 50.2 , 68.3 ) | 62.6 ( 56.4 , 68.9 ) | 62.6 ( 55.2 , 70.0 ) | 64.1 ( 53.5 , 74.8 ) | 67.2 ( 52.9 , 81.6 ) |
| Ethiopia 2016 | 1053 | 29.1 ( 24.7 , 33.5 ) | 32.0 ( 17.7 , 46.3 ) | 28.9 ( 24.4 , 33.4 ) | 38.4 ( 27.2 , 49.5 ) | 18.9 ( 9.2 , 28.7 ) | 22.1 ( 10.8 , 33.5 ) | 22.3 ( 8.8 , 35.9 ) | 30.2 ( 25.4 , 34.9 ) | 33.0 ( 26.7 , 39.4 ) | 26.0 ( 19.3 , 32.7 ) | 20.3 ( 9.6 , 31.0 ) |
| Gabon 2012 | 577 | 57.7 ( 50.2 , 65.2 ) | 70.3 ( 58.3 , 82.3 ) | 54.9 ( 46.5 , 63.2 ) | 60.7 ( 54.1 , 67.3 ) | 35.3 ( 19.0 , 51.7 ) | 53.8 ( 41.4 , 66.3 ) | 63.4 ( 56.2 , 70.6 ) | 61.1 ( 55.3 , 67.0 ) | 59.4 ( 33.6 , 85.1 ) | 61.6 ( 51.7 , 71.5 ) | 56.3 ( 46.7 , 65.9 ) |
| Gambia 2013 | 367 | 67.9 ( 61.9 , 73.9 ) | 65.3 ( 53.2 , 77.4 ) | 68.4 ( 61.3 , 75.5 ) | 73.9 ( 63.9 , 83.9 ) | 70.7 ( 54.7 , 86.6 ) | 73.1 ( 56.4 , 89.8 ) | 59.2 ( 46.5 , 71.8 ) | 73.4 ( 68.1 , 78.7 ) | 70.3 ( 62.6 , 78.1 ) | 71.7 ( 59.0 , 84.5 ) | 58.7 ( 44.9 , 72.4 ) |
| Ghana 2014 | 352 | 44.6 ( 38.5 , 50.7 ) | 64.0 ( 43.7 , 84.4 ) | 43.1 ( 36.7 , 49.4 ) | 66.6 ( 55.6 , 77.6 ) | 25.4 ( 14.3 , 36.6 ) | 28.4 ( 10.3 , 46.4 ) | 41.2 ( 30.5 , 51.8 ) | 52.1 ( 43.7 , 60.5 ) | 46.0 ( 34.4 , 57.6 ) | 68.6 ( 54.6 , 82.6 ) | 38.0 ( 30.4 , 45.6 ) |
| Guinea 2012 | 488 | 64.9 ( 60.4 , 69.4 ) | 69.2 ( 59.8 , 78.6 ) | 63.7 ( 58.3 , 69.0 ) | 67.3 ( 57.3 , 77.3 ) | 56.0 ( 42.7 , 69.2 ) | 56.1 ( 41.7 , 70.5 ) | 54.3 ( 42.1 , 66.5 ) | 68.3 ( 63.1 , 73.6 ) | 67.7 ( 62.6 , 72.7 ) | 63.8 ( 52.2 , 75.3 ) | 43.8 ( 28.0 , 59.5 ) |
| Kenya 2014 | | | | | | | | | | | | |
| Lesotho 2014 | 139 | 35.5 ( 26.8 , 44.3 ) | 28.4 ( 13.9 , 42.9 ) | 38.2 ( 27.1 , 49.2 ) | 43.2 ( 17.7 , 68.7 ) | | | 23.6 ( 5.5 , 41.7 ) | 31.3 ( 21.9 , 40.8 ) | | 34.4 ( 19.7 , 49.0 ) | 37.0 ( 24.9 , 49.2 ) |
| Liberia 2013 | | | | | | | | | | | | |
| Malawi 2015 | 632 | 45.1 ( 39.8 , 50.5 ) | 50.7 ( 41.2 , 60.2 ) | 43.3 ( 37.1 , 49.5 ) | 42.3 ( 32.1 , 52.4 ) | 49.2 ( 35.2 , 63.3 ) | | 44.1 ( 28.6 , 59.5 ) | 44.0 ( 38.6 , 49.5 ) | 58.0 ( 41.8 , 74.2 ) | 41.3 ( 35.7 , 46.9 ) | 49.6 ( 38.2 , 60.9 ) |
| Mali 2012 | 595 | 59.7 ( 54.5 , 64.9 ) | 53.5 ( 39.7 , 67.3 ) | 60.8 ( 55.4 , 66.1 ) | 71.5 ( 63.2 , 79.7 ) | 46.7 ( 33.4 , 60.1 ) | 52.6 ( 39.8 , 65.5 ) | 47.0 ( 33.0 , 60.9 ) | 61.4 ( 55.5 , 67.2 ) | 59.3 ( 53.6 , 65.0 ) | 67.1 ( 54.4 , 79.8 ) | 53.9 ( 38.2 , 69.5 ) |
| Mozambique 2011 | 1400 | 50.9 ( 47.3 , 54.4 ) | 49.5 ( 42.1 , 56.9 ) | 51.3 ( 47.6 , 54.9 ) | 53.6 ( 45.9 , 61.3 ) | 54.0 ( 46.8 , 61.1 ) | 51.6 ( 40.1 , 63.1 ) | 52.2 ( 46.3 , 58.1 ) | 50.4 ( 46.1 , 54.8 ) | 56.9 ( 51.0 , 62.7 ) | 46.7 ( 41.7 , 51.7 ) | 52.3 ( 44.3 , 60.3 ) |
| Namibia 2013 | 274 | 25.6 ( 19.5 , 31.7 ) | 25.7 ( 12.4 , 39.0 ) | 25.6 ( 18.7 , 32.5 ) | 31.5 ( 19.0 , 43.9 ) | 20.0 ( 3.7 , 36.3 ) | 24.2 ( 5.7 , 42.6 ) | 24.3 ( 16.4 , 32.2 ) | 27.6 ( 19.3 , 35.9 ) | 34.3 ( 12.2 , 56.5 ) | 18.1 ( 7.4 , 28.8 ) | 26.7 ( 19.2 , 34.2 ) |
| Niger 2012 | 681 | 58.6 ( 54.1 , 63.2 ) | 57.3 ( 44.9 , 69.8 ) | 58.9 ( 54.2 , 63.6 ) | 59.7 ( 44.7 , 74.6 ) | 53.0 ( 43.1 , 62.8 ) | 53.9 ( 37.4 , 70.5 ) | 47.2 ( 32.2 , 62.3 ) | 59.8 ( 54.9 , 64.7 ) | 59.4 ( 54.4 , 64.3 ) | 53.0 ( 41.6 , 64.5 ) | 62.0 ( 44.5 , 79.4 ) |
| Nigeria 2013 | | | | | | | | | | | | |
| Rwanda 2014 | 480 | 23.4 ( 19.4 , 27.3 ) | 26.0 ( 10.9 , 41.1 ) | 23.2 ( 19.1 , 27.3 ) | 28.0 ( 18.4 , 37.7 ) | 22.5 ( 14.3 , 30.7 ) | 25.3 ( 13.9 , 36.8 ) | 26.5 ( 15.7 , 37.3 ) | 22.9 ( 18.4 , 27.3 ) | 28.0 ( 15.3 , 40.7 ) | 21.9 ( 17.0 , 26.8 ) | 25.5 ( 15.6 , 35.3 ) |
| Senegal 2017 | 716 | 62.7 ( 57.9 , 67.5 ) | 73.3 ( 63.1 , 83.5 ) | 60.9 ( 55.7 , 66.0 ) | 66.5 ( 59.3 , 73.8 ) | 54.0 ( 39.7 , 68.2 ) | 51.8 ( 37.2 , 66.3 ) | 58.0 ( 51.0 , 64.9 ) | 67.4 ( 61.5 , 73.3 ) | 65.9 ( 60.0 , 71.9 ) | 60.5 ( 51.1 , 70.0 ) | 56.6 ( 45.9 , 67.3 ) |
| Sierra Leone 2013 | 667 | 54.0 ( 49.0 , 59.0 ) | 62.9 ( 52.8 , 73.1 ) | 52.0 ( 46.7 , 57.4 ) | 51.6 ( 41.6 , 61.6 ) | 45.7 ( 33.6 , 57.7 ) | 36.4 ( 17.7 , 55.1 ) | 49.0 ( 39.4 , 58.5 ) | 57.0 ( 51.1 , 62.9 ) | 56.5 ( 50.0 , 63.0 ) | 43.6 ( 31.8 , 55.3 ) | 53.2 ( 43.2 , 63.2 ) |
| South Africa 2016 | 106 | 39.1 ( 27.6 , 50.5 ) | | 37.4 ( 25.4 , 49.3 ) | 35.3 ( 11.5 , 59.1 ) | | 44.5 ( 23.0 , 66.1 ) | 29.1 ( 15.5 , 42.6 ) | 38.0 ( 23.4 , 52.5 ) | | | 40.2 ( 27.1 , 53.3 ) |
| Tanzania 2015 | 1124 | 57.1 ( 53.8 , 60.4 ) | 63.9 ( 55.9 , 71.9 ) | 55.3 ( 51.4 , 59.2 ) | 57.9 ( 51.2 , 64.6 ) | 50.2 ( 42.5 , 57.9 ) | 56.2 ( 42.2 , 70.2 ) | 52.7 ( 45.8 , 59.6 ) | 58.6 ( 54.6 , 62.6 ) | 63.4 ( 55.6 , 71.1 ) | 56.2 ( 52.1 , 60.4 ) | 53.9 ( 46.0 , 61.7 ) |
| Togo 2013 | 428 | 64.1 ( 59.2 , 69.0 ) | 78.6 ( 63.6 , 93.5 ) | 62.6 ( 57.4 , 67.8 ) | 60.9 ( 51.9 , 69.9 ) | 55.0 ( 43.5 , 66.6 ) | 68.4 ( 58.6 , 78.3 ) | 56.5 ( 42.6 , 70.5 ) | 63.8 ( 57.6 , 70.0 ) | 63.0 ( 55.2 , 70.8 ) | 69.3 ( 61.3 , 77.4 ) | 58.6 ( 47.9 , 69.2 ) |
| Uganda 2016 | 634 | 38.2 ( 33.7 , 42.7 ) | 41.3 ( 29.5 , 53.1 ) | 37.7 ( 32.7 , 42.6 ) | 39.5 ( 31.4 , 47.6 ) | 27.4 ( 17.3 , 37.6 ) | 36.3 ( 18.8 , 53.8 ) | 32.8 ( 20.3 , 45.2 ) | 39.5 ( 34.6 , 44.5 ) | 38.0 ( 24.2 , 51.8 ) | 39.5 ( 34.4 , 44.7 ) | 35.1 ( 26.7 , 43.5 ) |
| Zambia 2013 | | | | | | | | | | | | |
| Zimbabwe 2010 | 644 | 32.4 ( 28.5 , 36.4 ) | 39.2 ( 28.0 , 50.4 ) | 31.1 ( 27.2 , 35.0 ) | 35.0 ( 27.3 , 42.7 ) | 32.4 ( 22.1 , 42.7 ) | 29.7 ( 20.4 , 39.1 ) | 40.0 ( 28.8 , 51.1 ) | 31.7 ( 27.0 , 36.3 ) | | 30.8 ( 24.4 , 37.1 ) | 33.2 ( 28.0 , 38.4 ) |

Table S4: Prevalence of overweight by woman's age, wealth, residence and education using the latest surveys (2010-2017)

| Country, year | N | Overweight National % (95%CI) | Overweight by Woman's age % (95%CI) | | Overweight by Woman's Wealth % (95%CI) | | Overweight by Woman's Residence % (95%CI) | | | Overweight by Woman's Education % (95%CI) | | |
|---|---|---|---|---|---|---|---|---|---|---|---|---|
| | | | 15-19 years | 20-49 years | Poorest (Q1) | Richest (Q5) | Capital city | Other Urban | Rural | None | Primary | Secondary + |
| Benin 2017 | 6933 | 16.9 ( 15.9 , 18.0 ) | 8.3 ( 6.7 , 9.8 ) | 19.4 ( 18.2 , 20.6 ) | 9.7 ( 7.7 , 11.8 ) | 23.3 ( 21.0 , 25.7 ) | 24.4 ( 20.3 , 28.4 ) | 20.7 ( 18.9 , 22.5 ) | 13.7 ( 12.4 , 15.0 ) | 16.6 ( 15.2 , 18.0 ) | 17.5 ( 15.2 , 19.8 ) | 17.2 ( 15.4 , 19.1 ) |
| Burkina Faso 2010 | 7432 | 8.5 ( 7.7 , 9.3 ) | 5.9 ( 4.1 , 7.7 ) | 9.2 ( 8.2 , 10.1 ) | 3.5 ( 2.3 , 4.8 ) | 18.0 ( 15.8 , 20.1 ) | 19.3 ( 16.1 , 22.4 ) | 14.6 ( 12.3 , 16.8 ) | 5.3 ( 4.5 , 6.1 ) | 6.5 ( 5.7 , 7.3 ) | 12.0 ( 9.9 , 14.2 ) | 16.0 ( 13.6 , 18.5 ) |
| Burundi 2016 | 7714 | 6.5 ( 5.8 , 7.2 ) | 5.7 ( 4.2 , 7.1 ) | 6.8 ( 5.9 , 7.6 ) | 2.3 ( 1.4 , 3.2 ) | 15.6 ( 13.6 , 17.5 ) | 16.8 ( 13.5 , 20.1 ) | 15.3 ( 11.3 , 19.2 ) | 5.0 ( 4.3 , 5.7 ) | 3.9 ( 3.0 , 4.7 ) | 6.5 ( 5.4 , 7.6 ) | 10.0 ( 8.4 , 11.5 ) |
| Cameroon 2011 | 6991 | 22.2 ( 20.9 , 23.4 ) | 17.6 ( 15.3 , 19.8 ) | 23.7 ( 22.2 , 25.1 ) | 8.3 ( 6.0 , 10.5 ) | 29.1 ( 26.6 , 31.7 ) | 26.8 ( 24.2 , 29.4 ) | 24.6 ( 22.3 , 26.8 ) | 18.0 ( 16.2 , 19.9 ) | 11.2 ( 8.9 , 13.6 ) | 21.2 ( 19.3 , 23.0 ) | 27.0 ( 25.2 , 28.9 ) |
| Chad 2014 | 9426 | 9.5 ( 8.6 , 10.3 ) | 6.0 ( 4.7 , 7.3 ) | 10.5 ( 9.5 , 11.5 ) | 7.0 ( 5.5 , 8.5 ) | 17.8 ( 15.6 , 20.0 ) | 18.8 ( 14.5 , 23.0 ) | 16.2 ( 13.8 , 18.6 ) | 7.0 ( 6.1 , 7.9 ) | 7.3 ( 6.4 , 8.2 ) | 11.9 ( 9.9 , 14.0 ) | 14.2 ( 11.4 , 17.1 ) |
| Congo Brazzaville 2011 | 4927 | 17.7 ( 16.0 , 19.4 ) | 7.3 ( 4.9 , 9.7 ) | 20.6 ( 18.5 , 22.6 ) | 9.9 ( 8.4 , 11.4 ) | 27.3 ( 23.6 , 31.1 ) | 21.7 ( 18.3 , 25.1 ) | 20.0 ( 16.5 , 23.4 ) | 11.2 ( 10.0 , 12.5 ) | 13.7 ( 7.9 , 19.6 ) | 14.4 ( 11.6 , 17.2 ) | 19.2 ( 17.0 , 21.5 ) |
| Congo Democratic Republic 2013 | 7893 | 12.9 ( 11.5 , 14.4 ) | 7.9 ( 6.1 , 9.7 ) | 14.4 ( 12.7 , 16.2 ) | 6.9 ( 5.1 , 8.6 ) | 22.6 ( 20.2 , 25.1 ) | 23.5 ( 20.0 , 27.0 ) | 17.3 ( 14.9 , 19.7 ) | 9.1 ( 7.1 , 11.1 ) | 11.0 ( 8.5 , 13.5 ) | 11.4 ( 9.4 , 13.4 ) | 14.8 ( 12.8 , 16.7 ) |
| Cote d'Ivoire 2011 | 4195 | 20.0 ( 18.5 , 21.6 ) | 12.8 ( 9.8 , 15.9 ) | 22.0 ( 20.2 , 23.8 ) | 11.4 ( 8.5 , 14.4 ) | 27.6 ( 24.2 , 31.0 ) | 29.9 ( 25.8 , 34.0 ) | 22.1 ( 19.0 , 25.2 ) | 14.2 ( 12.4 , 16.0 ) | 18.5 ( 16.5 , 20.4 ) | 21.0 ( 18.0 , 24.0 ) | 22.9 ( 19.2 , 26.5 ) |
| Ethiopia 2016 | 13434 | 6.4 ( 5.7 , 7.1 ) | 4.9 ( 3.7 , 6.1 ) | 6.8 ( 6.0 , 7.6 ) | 2.6 ( 1.7 , 3.4 ) | 15.5 ( 13.7 , 17.2 ) | 22.5 ( 20.6 , 24.5 ) | 14.3 ( 11.4 , 17.2 ) | 3.4 ( 2.8 , 4.0 ) | 4.1 ( 3.4 , 4.7 ) | 6.5 ( 5.5 , 7.6 ) | 12.4 ( 10.5 , 14.4 ) |
| Gabon 2012 | 4841 | 25.7 ( 23.7 , 27.7 ) | 13.8 ( 10.4 , 17.3 ) | 28.9 ( 26.5 , 31.3 ) | 20.3 ( 17.5 , 23.1 ) | 27.5 ( 23.6 , 31.3 ) | 27.3 ( 24.1 , 30.4 ) | 23.8 ( 21.8 , 25.9 ) | 22.4 ( 19.6 , 25.3 ) | 29.4 ( 20.4 , 38.3 ) | 26.2 ( 23.4 , 29.0 ) | 25.3 ( 22.8 , 27.7 ) |
| Gambia 2013 | 4061 | 15.9 ( 14.5 , 17.2 ) | 9.2 ( 7.0 , 11.5 ) | 17.9 ( 16.3 , 19.5 ) | 11.5 ( 9.1 , 14.0 ) | 19.0 ( 15.6 , 22.4 ) | 24.2 ( 20.7 , 27.8 ) | 17.4 ( 15.3 , 19.5 ) | 13.6 ( 11.8 , 15.5 ) | 17.4 ( 15.3 , 19.5 ) | 16.0 ( 12.6 , 19.4 ) | 14.0 ( 11.6 , 16.4 ) |
| Ghana 2014 | 4314 | 25.3 ( 23.6 , 26.9 ) | 10.3 ( 7.9 , 12.7 ) | 28.6 ( 26.7 , 30.5 ) | 11.3 ( 8.9 , 13.7 ) | 32.4 ( 28.6 , 36.2 ) | 27.9 ( 23.4 , 32.4 ) | 29.2 ( 26.9 , 31.6 ) | 21.0 ( 18.6 , 23.5 ) | 18.2 ( 15.2 , 21.2 ) | 23.2 ( 19.7 , 26.6 ) | 28.0 ( 26.0 , 30.0 ) |
| Guinea 2012 | 4131 | 15.1 ( 13.7 , 16.4 ) | 10.2 ( 8.1 , 12.4 ) | 16.6 ( 14.9 , 18.3 ) | 4.7 ( 2.8 , 6.7 ) | 24.0 ( 21.0 , 26.9 ) | 24.6 ( 20.8 , 28.3 ) | 21.2 ( 18.3 , 24.2 ) | 10.4 ( 8.9 , 12.0 ) | 13.7 ( 12.1 , 15.3 ) | 14.8 ( 11.4 , 18.2 ) | 19.6 ( 16.5 , 22.6 ) |
| Kenya 2014 | 13213 | 23.3 ( 22.1 , 24.4 ) | 13.4 ( 11.4 , 15.5 ) | 25.5 ( 24.2 , 26.9 ) | 10.6 ( 9.1 , 12.1 ) | 31.0 ( 28.3 , 33.7 ) | 31.3 ( 25.6 , 37.0 ) | 28.2 ( 26.4 , 30.1 ) | 19.4 ( 18.0 , 20.8 ) | 11.9 ( 9.6 , 14.3 ) | 22.0 ( 20.5 , 23.5 ) | 26.5 ( 24.6 , 28.3 ) |
| Lesotho 2014 | 3193 | 25.6 ( 23.8 , 27.3 ) | 17.7 ( 14.1 , 21.4 ) | 27.8 ( 25.8 , 29.8 ) | 19.4 ( 16.2 , 22.7 ) | 28.3 ( 24.5 , 32.1 ) | 26.8 ( 20.3 , 33.3 ) | 28.7 ( 25.3 , 32.2 ) | 24.3 ( 22.4 , 26.2 ) | 18.7 ( 4.7 , 32.7 ) | 25.0 ( 22.3 , 27.7 ) | 26.1 ( 23.4 , 28.7 ) |
| Liberia 2013 | 4087 | 18.4 ( 16.8 , 20.0 ) | 9.3 ( 6.2 , 12.5 ) | 20.9 ( 19.0 , 22.9 ) | 15.1 ( 12.4 , 17.8 ) | 21.6 ( 17.5 , 25.7 ) | 20.7 ( 17.5 , 24.0 ) | 17.6 ( 14.8 , 20.5 ) | 16.7 ( 14.8 , 18.5 ) | 19.8 ( 17.2 , 22.3 ) | 15.7 ( 13.0 , 18.3 ) | 19.4 ( 16.6 , 22.3 ) |
| Malawi 2015 | 7275 | 15.6 ( 14.5 , 16.6 ) | 8.6 ( 6.9 , 10.3 ) | 17.5 ( 16.2 , 18.7 ) | 10.3 ( 7.7 , 12.9 ) | 23.5 ( 21.0 , 26.0 ) | 21.0 ( 17.7 , 24.2 ) | 24.8 ( 21.1 , 28.6 ) | 13.8 ( 12.6 , 14.9 ) | 17.9 ( 14.7 , 21.2 ) | 13.4 ( 12.1 , 14.6 ) | 19.5 ( 17.4 , 21.7 ) |
| Mali 2012 | 4509 | 13.4 ( 12.2 , 14.7 ) | 8.0 ( 5.9 , 10.1 ) | 14.6 ( 13.2 , 16.0 ) | 11.0 ( 8.5 , 13.6 ) | 21.2 ( 18.8 , 23.5 ) | 21.0 ( 18.5 , 23.4 ) | 19.2 ( 16.0 , 22.4 ) | 11.1 ( 9.6 , 12.6 ) | 12.6 ( 11.1 , 14.1 ) | 17.3 ( 13.6 , 21.0 ) | 14.9 ( 12.3 , 17.5 ) |
| Mozambique 2011 | 11877 | 12.9 ( 12.1 , 13.7 ) | 9.0 ( 7.8 , 10.3 ) | 14.0 ( 13.1 , 14.9 ) | 5.0 ( 3.4 , 6.6 ) | 24.3 ( 22.7 , 25.9 ) | 26.0 ( 23.4 , 28.5 ) | 17.4 ( 15.6 , 19.2 ) | 9.5 ( 8.5 , 10.5 ) | 9.0 ( 7.8 , 10.1 ) | 12.8 ( 11.6 , 14.0 ) | 19.9 ( 18.1 , 21.8 ) |
| Namibia 2013 | 4008 | 18.8 ( 17.4 , 20.3 ) | 7.8 ( 5.5 , 10.0 ) | 21.7 ( 20.0 , 23.5 ) | 10.6 ( 7.9 , 13.2 ) | 21.7 ( 18.2 , 25.1 ) | 23.2 ( 18.8 , 27.5 ) | 22.2 ( 20.2 , 24.3 ) | 14.4 ( 12.6 , 16.1 ) | 19.7 ( 13.1 , 26.4 ) | 17.2 ( 14.5 , 20.0 ) | 19.2 ( 17.6 , 20.8 ) |
| Niger 2012 | 4243 | 14.1 ( 12.8 , 15.4 ) | 4.8 ( 3.3 , 6.3 ) | 16.0 ( 14.5 , 17.5 ) | 8.9 ( 6.2 , 11.5 ) | 25.8 ( 23.2 , 28.5 ) | 28.2 ( 24.4 , 31.9 ) | 24.5 ( 20.8 , 28.2 ) | 11.3 ( 9.8 , 12.8 ) | 13.2 ( 11.7 , 14.7 ) | 16.6 ( 13.3 , 19.8 ) | 19.1 ( 15.2 , 23.1 ) |
| Nigeria 2013 | 33063 | 17.6 ( 16.9 , 18.3 ) | 6.9 ( 6.2 , 7.7 ) | 20.5 ( 19.7 , 21.3 ) | 8.5 ( 7.4 , 9.6 ) | 26.1 ( 24.7 , 27.4 ) | 26.4 ( 23.3 , 29.4 ) | 21.6 ( 20.5 , 22.7 ) | 14.1 ( 13.3 , 14.9 ) | 12.2 ( 11.2 , 13.1 ) | 20.5 ( 19.2 , 21.9 ) | 20.7 ( 19.8 , 21.6 ) |
| Rwanda 2014 | 6108 | 17.8 ( 16.8 , 18.9 ) | 16.1 ( 14.0 , 18.2 ) | 18.3 ( 17.1 , 19.5 ) | 10.7 ( 8.7 , 12.6 ) | 26.4 ( 23.9 , 28.9 ) | 27.2 ( 23.8 , 30.6 ) | 27.3 ( 22.9 , 31.6 ) | 15.5 ( 14.4 , 16.5 ) | 13.7 ( 11.1 , 16.3 ) | 17.1 ( 15.9 , 18.3 ) | 21.8 ( 19.7 , 23.9 ) |
| Senegal 2010 | 5128 | 15.7 ( 13.9 , 17.5 ) | 7.5 ( 5.0 , 10.0 ) | 18.3 ( 16.1 , 20.5 ) | 10.2 ( 8.0 , 12.4 ) | 21.3 ( 17.1 , 25.6 ) | 24.4 ( 18.9 , 29.8 ) | 16.3 ( 14.6 , 18.1 ) | 10.9 ( 9.6 , 12.2 ) | 14.9 ( 12.9 , 16.9 ) | 18.4 ( 15.1 , 21.7 ) | 15.2 ( 11.4 , 19.0 ) |
| Sierra Leone 2013 | 7162 | 14.0 ( 12.8 , 15.2 ) | 8.8 ( 7.1 , 10.5 ) | 15.4 ( 14.0 , 16.8 ) | 8.5 ( 6.7 , 10.2 ) | 19.4 ( 16.8 , 22.1 ) | 17.4 ( 13.6 , 21.2 ) | 19.6 ( 17.5 , 21.7 ) | 11.3 ( 9.9 , 12.6 ) | 13.9 ( 12.3 , 15.6 ) | 12.7 ( 10.0 , 15.3 ) | 14.6 ( 12.6 , 16.6 ) |
| South Africa 2016 | 3210 | 26.8 ( 25.1 , 28.6 ) | 19.4 ( 15.3 , 23.5 ) | 28.3 ( 26.2 , 30.3 ) | 27.8 ( 23.9 , 31.7 ) | 23.6 ( 18.4 , 28.8 ) | 25.4 ( 21.9 , 28.9 ) | 27.9 ( 24.8 , 31.0 ) | 27.5 ( 25.1 , 29.9 ) | 18.0 ( 6.9 , 29.1 ) | 27.8 ( 21.4 , 34.1 ) | 26.9 ( 25.1 , 28.8 ) |
| Tanzania 2015 | 11735 | 19.0 ( 18.0 , 20.1 ) | 12.0 ( 10.3 , 13.8 ) | 21.0 ( 19.9 , 22.2 ) | 10.5 ( 8.9 , 12.2 ) | 26.4 ( 24.2 , 28.5 ) | 26.6 ( 22.6 , 30.5 ) | 23.7 ( 21.9 , 25.5 ) | 15.8 ( 14.6 , 16.9 ) | 16.3 ( 14.2 , 18.4 ) | 18.7 ( 17.5 , 19.9 ) | 21.5 ( 19.0 , 24.1 ) |
| Togo 2013 | 4326 | 20.1 ( 18.6 , 21.6 ) | 12.6 ( 10.0 , 15.1 ) | 21.8 ( 20.1 , 23.6 ) | 8.6 ( 6.5 , 10.7 ) | 27.5 ( 24.0 , 31.0 ) | 27.8 ( 24.9 , 30.7 ) | 20.9 ( 16.3 , 25.5 ) | 15.3 ( 13.5 , 17.0 ) | 16.2 ( 13.8 , 18.6 ) | 22.5 ( 19.9 , 25.0 ) | 21.0 ( 18.5 , 23.5 ) |
| Uganda 2016 | 5240 | 17.1 ( 15.8 , 18.3 ) | 12.2 ( 10.0 , 14.4 ) | 18.6 ( 17.1 , 20.1 ) | 7.3 ( 5.6 , 9.0 ) | 25.6 ( 23.0 , 28.1 ) | 26.7 ( 21.7 , 31.8 ) | 21.5 ( 18.5 , 24.5 ) | 15.1 ( 13.6 , 16.5 ) | 15.9 ( 12.3 , 19.4 ) | 14.6 ( 13.2 , 16.0 ) | 21.6 ( 19.0 , 24.1 ) |
| Zambia 2013 | 14510 | 16.6 ( 15.8 , 17.4 ) | 9.5 ( 8.2 , 10.7 ) | 18.7 ( 17.7 , 19.6 ) | 8.2 ( 7.0 , 9.4 ) | 24.1 ( 22.3 , 25.9 ) | 22.9 ( 20.4 , 25.4 ) | 21.1 ( 19.8 , 22.4 ) | 11.9 ( 10.9 , 12.8 ) | 15.1 ( 12.6 , 17.6 ) | 14.6 ( 13.5 , 15.6 ) | 18.9 ( 17.6 , 20.1 ) |
| Zimbabwe 2010 | 7931 | 21.3 ( 20.3 , 22.2 ) | 13.6 ( 11.8 , 15.4 ) | 23.4 ( 22.2 , 24.6 ) | 13.8 ( 11.7 , 15.8 ) | 26.3 ( 24.1 , 28.4 ) | 26.6 ( 23.7 , 29.5 ) | 24.9 ( 22.9 , 26.9 ) | 18.5 ( 17.3 , 19.6 ) | 25.0 ( 18.2 , 31.8 ) | 19.5 ( 17.7 , 21.4 ) | 21.8 ( 20.6 , 23.0 ) |

Table S5: Prevalence of obesity by woman's age, wealth, residence and education using the latest surveys (2010-2017)

| Country, year | N | Obesity National % (95%CI) | Obesity by Woman's age % (95%CI) | | Obesity by Woman's Wealth % (95%CI) | | Obesity by Woman's Residence % (95%CI) | | | Obesity by Woman's Education % (95%CI) | | |
|---|---|---|---|---|---|---|---|---|---|---|---|---|
| | | | 15-19 years | 20-49 years | Poorest (Q1) | Richest (Q5) | Capital city | Other Urban | Rural | None | Primary | Secondary + |
| Benin 2017 | 6933 | 9.5 ( 8.6 , 10.4 ) | 1.0 ( 0.5 , 1.5 ) | 11.8 ( 10.7 , 12.9 ) | 2.0 ( 1.1 , 2.9 ) | 20.1 ( 17.9 , 22.4 ) | 24.4 ( 20.6 , 28.3 ) | 12.6 ( 10.8 , 14.5 ) | 5.8 ( 4.9 , 6.7 ) | 8.3 ( 7.1 , 9.4 ) | 12.9 ( 10.8 , 14.9 ) | 9.4 ( 7.9 , 10.9 ) |
| Burkina Faso 2010 | 7432 | 3.1 ( 2.6 , 3.5 ) | 0.6 ( 0.1 , 1.0 ) | 3.7 ( 3.1 , 4.3 ) | 0.2 ( 0.0 , 0.5 ) | 9.5 ( 8.0 , 11.0 ) | 11.0 ( 8.4 , 13.6 ) | 6.3 ( 5.1 , 7.5 ) | 0.9 ( 0.6 , 1.2 ) | 1.9 ( 1.4 , 2.3 ) | 4.6 ( 3.0 , 6.3 ) | 8.0 ( 6.0 , 9.9 ) |
| Burundi 2016 | 7714 | 1.8 ( 1.4 , 2.2 ) | 0.4 ( 0.1 , 0.7 ) | 2.2 ( 1.7 , 2.7 ) | 0.1 ( -0.1 , 0.2 ) | 7.2 ( 5.7 , 8.7 ) | 11.2 ( 7.6 , 14.8 ) | 7.8 ( 5.0 , 10.5 ) | 0.5 ( 0.3 , 0.8 ) | 0.6 ( 0.3 , 0.9 ) | 1.3 ( 0.8 , 1.8 ) | 4.0 ( 2.8 , 5.2 ) |
| Cameroon 2011 | 6991 | 10.9 ( 10.1 , 11.7 ) | 2.3 ( 1.6 , 3.1 ) | 13.7 ( 12.7 , 14.7 ) | 0.9 ( 0.4 , 1.4 ) | 19.0 ( 17.1 , 20.8 ) | 20.0 ( 17.7 , 22.2 ) | 12.7 ( 11.3 , 14.2 ) | 4.8 ( 4.0 , 5.7 ) | 3.8 ( 2.6 , 5.0 ) | 11.6 ( 10.2 , 13.0 ) | 13.1 ( 11.8 , 14.4 ) |
| Chad 2014 | 9426 | 2.5 ( 2.1 , 2.9 ) | 0.6 ( 0.2 , 1.0 ) | 3.0 ( 2.5 , 3.6 ) | 1.1 ( 0.5 , 1.7 ) | 7.4 ( 6.2 , 8.5 ) | 10.1 ( 8.2 , 12.0 ) | 5.5 ( 4.0 , 7.0 ) | 1.0 ( 0.7 , 1.2 ) | 1.7 ( 1.3 , 2.0 ) | 3.3 ( 2.1 , 4.5 ) | 4.5 ( 3.2 , 5.8 ) |
| Congo Brazzaville 2011 | 4927 | 8.9 ( 7.6 , 10.3 ) | 1.0 ( 0.1 , 1.9 ) | 11.1 ( 9.4 , 12.7 ) | 2.2 ( 1.5 , 2.9 ) | 15.4 ( 12.1 , 18.7 ) | 11.0 ( 8.1 , 13.9 ) | 11.7 ( 9.3 , 14.0 ) | 3.8 ( 2.4 , 5.2 ) | 4.1 ( 1.3 , 6.8 ) | 6.1 ( 3.7 , 8.4 ) | 10.3 ( 8.4 , 12.2 ) |
| Congo Democratic Republic 2013 | 7893 | 3.4 ( 2.8 , 4.1 ) | 1.5 ( 0.5 , 2.5 ) | 4.0 ( 3.2 , 4.8 ) | 0.4 ( 0.1 , 0.8 ) | 10.2 ( 8.6 , 11.8 ) | 9.0 ( 6.4 , 11.6 ) | 6.2 ( 4.9 , 7.5 ) | 1.2 ( 0.5 , 1.9 ) | 2.1 ( 0.5 , 3.7 ) | 1.8 ( 1.2 , 2.5 ) | 5.1 ( 4.1 , 6.0 ) |
| Cote d'Ivoire 2011 | 4195 | 6.5 ( 5.4 , 7.6 ) | 1.2 ( 0.4 , 1.9 ) | 8.0 ( 6.7 , 9.3 ) | 1.3 ( 0.3 , 2.4 ) | 10.6 ( 7.6 , 13.6 ) | 10.3 ( 7.4 , 13.2 ) | 9.9 ( 7.4 , 12.4 ) | 2.8 ( 1.9 , 3.7 ) | 6.4 ( 4.9 , 7.8 ) | 7.3 ( 5.5 , 9.2 ) | 5.9 ( 3.9 , 8.0 ) |
| Ethiopia 2016 | 13434 | 1.6 ( 1.1 , 2.0 ) | 0.3 ( 0.1 , 0.5 ) | 1.9 ( 1.4 , 2.5 ) | 0.2 ( 0.1 , 0.3 ) | 5.0 ( 3.5 , 6.4 ) | 7.9 ( 6.6 , 9.3 ) | 4.9 ( 2.6 , 7.1 ) | 0.4 ( 0.2 , 0.5 ) | 0.6 ( 0.4 , 0.9 ) | 1.6 ( 1.0 , 2.2 ) | 4.1 ( 2.7 , 5.4 ) |
| Gabon 2012 | 4841 | 18.9 ( 16.9 , 20.8 ) | 4.2 ( 2.2 , 6.3 ) | 22.9 ( 20.5 , 25.3 ) | 9.5 ( 7.4 , 11.6 ) | 23.3 ( 19.1 , 27.4 ) | 22.3 ( 19.3 , 25.3 ) | 14.9 ( 13.1 , 16.8 ) | 11.8 ( 9.1 , 14.5 ) | 28.5 ( 19.6 , 37.5 ) | 19.6 ( 16.4 , 22.9 ) | 18.0 ( 15.8 , 20.3 ) |
| Gambia 2013 | 4061 | 7.3 ( 6.2 , 8.5 ) | 2.1 ( 1.0 , 3.3 ) | 9.0 ( 7.5 , 10.4 ) | 4.2 ( 2.3 , 6.1 ) | 11.6 ( 8.0 , 15.1 ) | 16.5 ( 12.6 , 20.4 ) | 9.9 ( 8.1 , 11.8 ) | 3.8 ( 2.8 , 4.7 ) | 6.5 ( 5.2 , 7.9 ) | 6.2 ( 3.2 , 9.2 ) | 8.6 ( 6.4 , 10.8 ) |
| Ghana 2014 | 4314 | 15.4 ( 13.8 , 17.0 ) | 1.5 ( 0.5 , 2.5 ) | 18.5 ( 16.6 , 20.3 ) | 1.7 ( 0.8 , 2.7 ) | 29.0 ( 25.6 , 32.3 ) | 29.9 ( 24.5 , 35.3 ) | 16.3 ( 13.9 , 18.8 ) | 8.7 ( 7.2 , 10.1 ) | 8.8 ( 6.4 , 11.2 ) | 15.2 ( 11.8 , 18.5 ) | 17.4 ( 15.5 , 19.3 ) |
| Guinea 2012 | 4131 | 4.9 ( 4.0 , 5.8 ) | 1.2 ( 0.3 , 2.1 ) | 6.1 ( 4.9 , 7.2 ) | 1.1 ( 0.3 , 1.8 ) | 11.6 ( 8.9 , 14.2 ) | 12.0 ( 9.1 , 14.9 ) | 6.6 ( 4.7 , 8.6 ) | 2.2 ( 1.4 , 2.9 ) | 3.5 ( 2.6 , 4.4 ) | 5.9 ( 4.0 , 7.8 ) | 8.5 ( 5.2 , 11.9 ) |
| Kenya 2014 | 13213 | 10.1 ( 9.4 , 10.9 ) | 1.8 ( 1.1 , 2.5 ) | 12.1 ( 11.2 , 13.0 ) | 1.9 ( 1.3 , 2.4 ) | 19.4 ( 17.2 , 21.6 ) | 16.5 ( 12.5 , 20.5 ) | 14.0 ( 12.5 , 15.5 ) | 7.1 ( 6.4 , 7.8 ) | 5.8 ( 3.9 , 7.6 ) | 9.1 ( 8.1 , 10.0 ) | 12.1 ( 10.8 , 13.3 ) |
| Lesotho 2014 | 3193 | 20.0 ( 17.9 , 22.0 ) | 4.7 ( 2.7 , 6.7 ) | 24.2 ( 21.8 , 26.6 ) | 6.2 ( 4.1 , 8.3 ) | 27.3 ( 23.0 , 31.7 ) | 21.8 ( 13.7 , 30.0 ) | 23.7 ( 20.0 , 27.5 ) | 18.3 ( 16.2 , 20.5 ) | 19.1 ( -0.7 , 39.0 ) | 18.6 ( 15.9 , 21.2 ) | 20.9 ( 18.4 , 23.4 ) |
| Liberia 2013 | 4087 | 8.6 ( 7.2 , 10.1 ) | 0.7 ( -0.2 , 1.5 ) | 10.9 ( 9.0 , 12.7 ) | 3.4 ( 2.2 , 4.5 ) | 14.6 ( 10.4 , 18.8 ) | 12.7 ( 9.4 , 16.0 ) | 7.3 ( 5.7 , 8.9 ) | 5.4 ( 4.5 , 6.4 ) | 10.0 ( 7.8 , 12.1 ) | 5.7 ( 4.0 , 7.3 ) | 9.8 ( 7.0 , 12.7 ) |
| Malawi 2015 | 7275 | 5.7 ( 5.0 , 6.4 ) | 1.4 ( 0.6 , 2.2 ) | 6.9 ( 6.1 , 7.7 ) | 1.7 ( 0.4 , 2.9 ) | 13.1 ( 11.3 , 15.0 ) | 14.0 ( 10.7 , 17.2 ) | 13.2 ( 10.4 , 15.9 ) | 4.0 ( 3.3 , 4.6 ) | 5.0 ( 3.2 , 6.7 ) | 4.4 ( 3.6 , 5.1 ) | 9.1 ( 7.4 , 10.7 ) |
| Mali 2012 | 4509 | 5.2 ( 4.5 , 5.9 ) | 1.7 ( 0.7 , 2.6 ) | 5.9 ( 5.1 , 6.8 ) | 1.2 ( 0.4 , 1.9 ) | 13.7 ( 11.8 , 15.6 ) | 15.6 ( 13.1 , 18.2 ) | 10.9 ( 8.4 , 13.4 ) | 2.3 ( 1.7 , 3.0 ) | 3.8 ( 3.1 , 4.5 ) | 10.7 ( 7.7 , 13.8 ) | 8.4 ( 6.2 , 10.6 ) |
| Mozambique 2011 | 11877 | 4.2 ( 3.7 , 4.7 ) | 0.8 ( 0.5 , 1.2 ) | 5.2 ( 4.6 , 5.8 ) | 0.3 ( 0.0 , 0.6 ) | 13.0 ( 11.7 , 14.2 ) | 15.0 ( 13.2 , 16.9 ) | 7.4 ( 6.2 , 8.7 ) | 1.6 ( 1.2 , 2.0 ) | 1.7 ( 1.2 , 2.2 ) | 3.9 ( 3.3 , 4.4 ) | 9.5 ( 8.1 , 11.0 ) |
| Namibia 2013 | 4008 | 13.3 ( 12.0 , 14.6 ) | 2.3 ( 1.0 , 3.5 ) | 16.2 ( 14.6 , 17.8 ) | 2.3 ( 1.1 , 3.6 ) | 22.8 ( 19.8 , 25.8 ) | 18.5 ( 14.0 , 23.0 ) | 17.8 ( 15.8 , 19.8 ) | 7.6 ( 6.3 , 8.9 ) | 15.4 ( 9.3 , 21.4 ) | 8.6 ( 6.5 , 10.7 ) | 14.4 ( 12.9 , 15.9 ) |
| Niger 2012 | 4243 | 3.9 ( 3.2 , 4.6 ) | 0.9 ( -0.1 , 1.9 ) | 4.5 ( 3.7 , 5.2 ) | 1.8 ( 0.0 , 3.6 ) | 11.6 ( 9.8 , 13.5 ) | 15.9 ( 13.3 , 18.4 ) | 9.9 ( 6.7 , 13.0 ) | 1.9 ( 1.2 , 2.5 ) | 2.9 ( 2.3 , 3.6 ) | 8.1 ( 5.4 , 10.7 ) | 7.0 ( 4.6 , 9.5 ) |
| Nigeria 2013 | 33063 | 7.6 ( 7.1 , 8.0 ) | 1.4 ( 1.0 , 1.8 ) | 9.2 ( 8.6 , 9.8 ) | 1.9 ( 1.1 , 2.7 ) | 16.0 ( 15.0 , 17.1 ) | 18.5 ( 16.3 , 20.8 ) | 10.5 ( 9.7 , 11.3 ) | 4.5 ( 4.0 , 5.1 ) | 3.6 ( 3.0 , 4.2 ) | 8.9 ( 7.9 , 9.9 ) | 10.1 ( 9.4 , 10.8 ) |
| Rwanda 2014 | 6108 | 3.8 ( 3.3 , 4.3 ) | 1.1 ( 0.6 , 1.7 ) | 4.6 ( 4.0 , 5.2 ) | 0.5 ( 0.1 , 0.9 ) | 11.2 ( 9.5 , 12.8 ) | 12.0 ( 9.3 , 14.7 ) | 9.0 ( 6.8 , 11.2 ) | 2.1 ( 1.7 , 2.6 ) | 2.6 ( 1.3 , 3.9 ) | 3.0 ( 2.5 , 3.6 ) | 6.6 ( 5.3 , 7.9 ) |
| Senegal 2010 | 5128 | 6.0 ( 5.0 , 6.9 ) | 1.0 ( 0.3 , 1.7 ) | 7.5 ( 6.2 , 8.7 ) | 2.6 ( 1.6 , 3.6 ) | 9.6 ( 6.7 , 12.5 ) | 9.4 ( 6.1 , 12.7 ) | 8.1 ( 6.5 , 9.8 ) | 3.1 ( 2.5 , 3.7 ) | 5.7 ( 4.5 , 6.9 ) | 7.5 ( 5.4 , 9.5 ) | 5.2 ( 3.1 , 7.3 ) |
| Sierra Leone 2013 | 7162 | 5.1 ( 4.3 , 5.9 ) | 2.0 ( 1.1 , 2.9 ) | 6.0 ( 5.1 , 6.8 ) | 2.0 ( 1.1 , 2.9 ) | 11.1 ( 9.1 , 13.1 ) | 11.1 ( 8.4 , 13.9 ) | 7.8 ( 6.2 , 9.3 ) | 2.6 ( 2.0 , 3.2 ) | 4.1 ( 3.3 , 5.0 ) | 4.9 ( 3.3 , 6.4 ) | 6.9 ( 5.4 , 8.3 ) |
| South Africa 2016 | 3210 | 36.1 ( 33.8 , 38.5 ) | 12.3 ( 8.9 , 15.8 ) | 40.8 ( 38.2 , 43.4 ) | 27.7 ( 23.4 , 32.0 ) | 42.8 ( 36.0 , 49.7 ) | 36.2 ( 31.5 , 40.9 ) | 37.8 ( 34.5 , 41.1 ) | 34.9 ( 31.2 , 38.7 ) | 41.6 ( 27.3 , 56.0 ) | 36.6 ( 29.6 , 43.7 ) | 35.9 ( 33.5 , 38.4 ) |
| Tanzania 2015 | 11735 | 10.1 ( 9.2 , 11.0 ) | 2.0 ( 1.3 , 2.7 ) | 12.4 ( 11.3 , 13.5 ) | 1.8 ( 1.2 , 2.4 ) | 21.7 ( 19.8 , 23.6 ) | 21.3 ( 17.6 , 24.9 ) | 16.4 ( 14.3 , 18.5 ) | 5.5 ( 4.8 , 6.3 ) | 4.9 ( 3.6 , 6.1 ) | 10.1 ( 9.0 , 11.1 ) | 13.3 ( 11.5 , 15.2 ) |
| Togo 2013 | 4326 | 11.2 ( 10.1 , 12.3 ) | 2.0 ( 0.9 , 3.1 ) | 13.4 ( 12.1 , 14.7 ) | 2.5 ( 1.3 , 3.8 ) | 21.6 ( 18.8 , 24.4 ) | 19.1 ( 16.5 , 21.7 ) | 15.5 ( 12.6 , 18.4 ) | 5.4 ( 4.3 , 6.4 ) | 9.0 ( 7.3 , 10.7 ) | 12.8 ( 10.8 , 14.9 ) | 11.5 ( 9.7 , 13.3 ) |
| Uganda 2016 | 5240 | 7.3 ( 6.3 , 8.3 ) | 1.3 ( 0.6 , 2.0 ) | 9.2 ( 8.0 , 10.4 ) | 0.8 ( 0.2 , 1.4 ) | 17.0 ( 14.6 , 19.4 ) | 17.1 ( 12.7 , 21.5 ) | 11.4 ( 9.2 , 13.6 ) | 5.4 ( 4.3 , 6.5 ) | 6.2 ( 3.2 , 9.2 ) | 5.4 ( 4.3 , 6.4 ) | 10.9 ( 9.2 , 12.5 ) |
| Zambia 2013 | 14510 | 6.7 ( 6.2 , 7.3 ) | 1.4 ( 0.9 , 1.9 ) | 8.3 ( 7.6 , 9.0 ) | 0.8 ( 0.4 , 1.2 ) | 13.7 ( 12.0 , 15.3 ) | 13.3 ( 11.4 , 15.3 ) | 9.4 ( 8.2 , 10.5 ) | 3.0 ( 2.5 , 3.5 ) | 3.2 ( 2.0 , 4.5 ) | 5.2 ( 4.5 , 5.9 ) | 8.9 ( 7.9 , 9.9 ) |
| Zimbabwe 2010 | 7931 | 10.7 ( 9.9 , 11.6 ) | 2.3 ( 1.5 , 3.1 ) | 13.1 ( 12.0 , 14.2 ) | 4.1 ( 2.8 , 5.3 ) | 17.0 ( 15.0 , 18.9 ) | 17.4 ( 14.5 , 20.2 ) | 14.3 ( 12.6 , 16.0 ) | 7.6 ( 6.6 , 8.6 ) | 10.4 ( 4.3 , 16.5 ) | 9.7 ( 8.4 , 10.9 ) | 11.2 ( 10.1 , 12.3 ) |

Figures S2 A-E: Regional trend in prevalence (%) of underweight (A), anemia (B), anemia during pregnancy (C), overweight (D) and obesity (E) according to women's age (3 categories: 15-19 years, 20-34 years, 35-49 years)

A

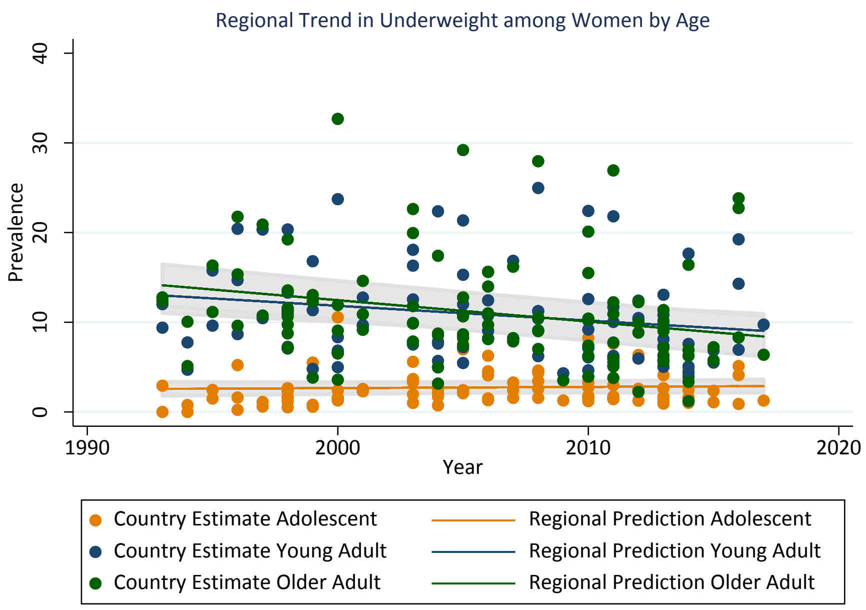


B

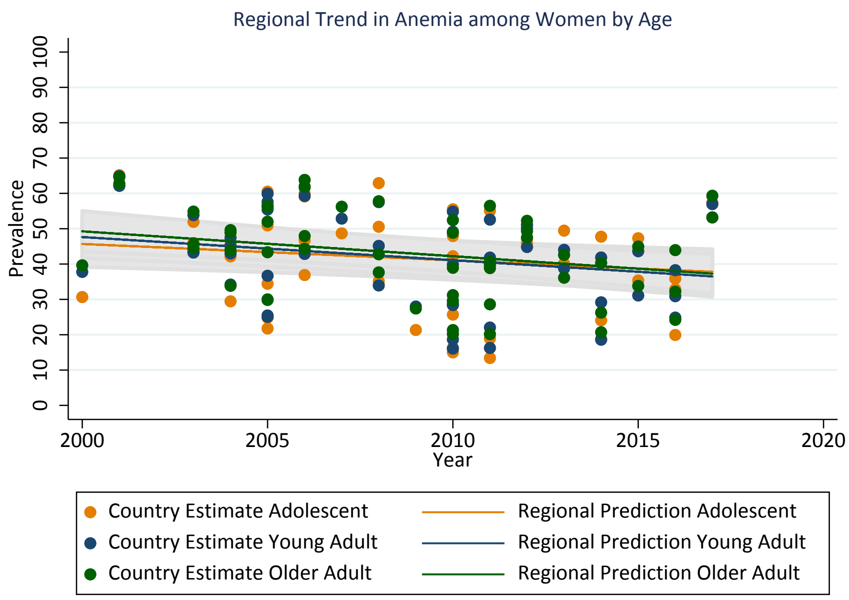

C

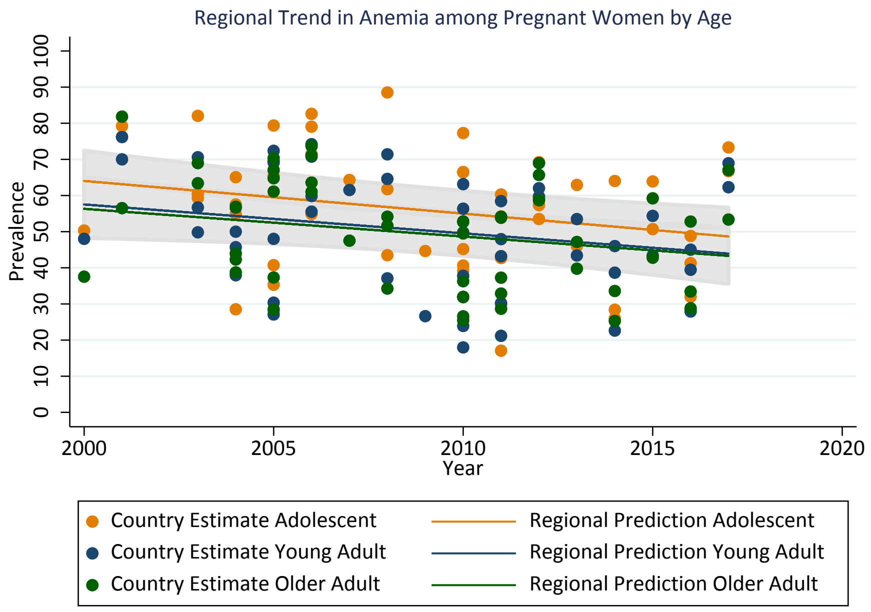
Regional Trend in Anemia among Pregnant Women by Age
Prevalence
0
10
20
30
40
50
60
70
80
90
100
2000
2005
2010
2015
2020
Year
Country Estimate Adolescent
Country Estimate Young Adult
Country Estimate Older Adult
Regional Prediction Adolescent
Regional Prediction Young Adult
Regional Prediction Older Adult

D

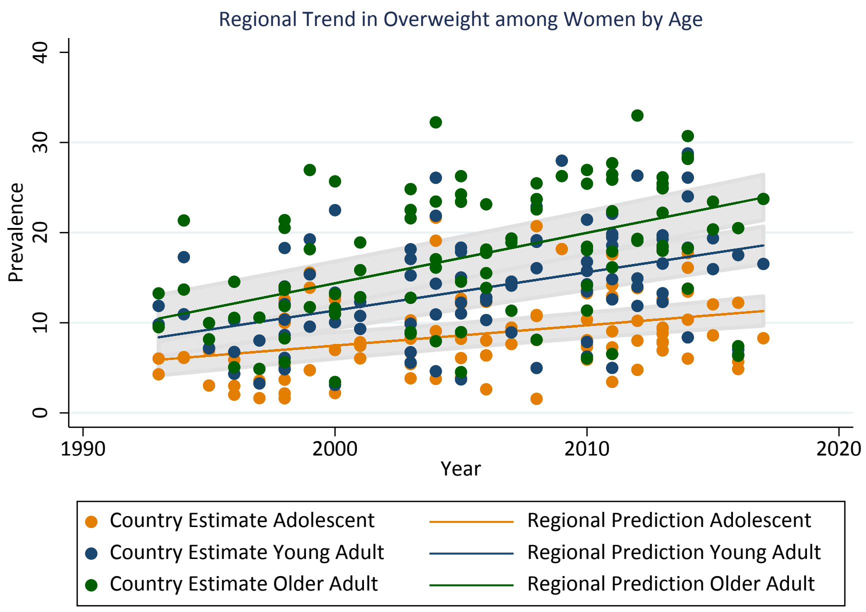
Regional Trend in Overweight among Women by Age
Prevalence
0
10
20
30
40
1990
2000
2010
2020
Year
Country Estimate Adolescent
Country Estimate Young Adult
Country Estimate Older Adult
Regional Prediction Adolescent
Regional Prediction Young Adult
Regional Prediction Older Adult

E

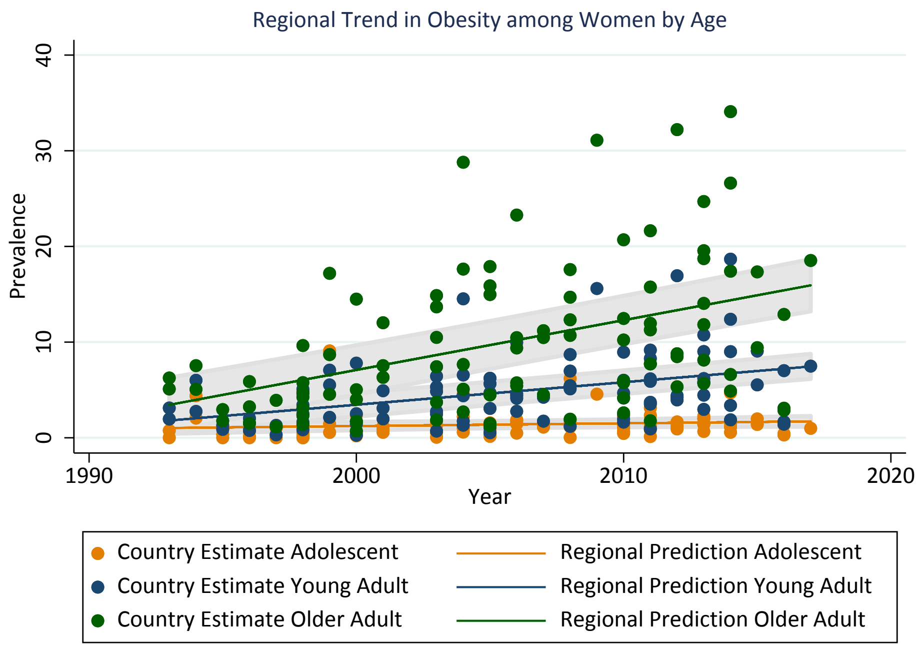
Regional Trend in Obesity among Women by Age
Prevalence
0
10
20
30
40
1990
2000
2010
2020
Year
Country Estimate Adolescent
Country Estimate Young Adult
Country Estimate Older Adult
Regional Prediction Adolescent
Regional Prediction Young Adult
Regional Prediction Older Adult
